# Prevalence of sexually transmitted infections among young people in South Africa: A nested survey in a health and demographic surveillance site

**DOI:** 10.1371/journal.pmed.1002512

**Published:** 2018-02-27

**Authors:** Suzanna C. Francis, T. Nondumiso Mthiyane, Kathy Baisley, S. Lerato Mchunu, Jane B. Ferguson, Theresa Smit, Tania Crucitti, Dickman Gareta, Siphephelo Dlamini, Tinofa Mutevedzi, Janet Seeley, Deenan Pillay, Nuala McGrath, Maryam Shahmanesh

**Affiliations:** 1 MRC Tropical Epidemiology Group, London School of Hygiene & Tropical Medicine, London, United Kingdom; 2 Africa Health Research Institute, KwaZulu-Natal, South Africa; 3 Centre for Maternal, Adolescent, Reproductive, and Child Health, London School of Hygiene & Tropical Medicine, London, United Kingdom; 4 HIV/STI Reference Laboratory, Institute of Tropical Medicine, Antwerp, Belgium; 5 Department of Global Health and Development, London School of Hygiene & Tropical Medicine, London, United Kingdom; 6 Africa Health Research Institute, School of Nursing & Public Health, University of KwaZulu-Natal, KwaZulu-Natal, South Africa; 7 Division of Infection and Immunity, University College London, London, United Kingdom; 8 Academic Unit of Primary Care and Population Sciences, University of Southampton, Southampton, United Kingdom; 9 Department of Social Statistics and Demography, University of Southampton, Southampton, United Kingdom; 10 Research Department of Epidemiology & Public Health, University College London, London, United Kingdom; 11 Institute for Global Health, University College London, London, United Kingdom; University of Bern, SWITZERLAND

## Abstract

**Background:**

Sexually transmitted infections (STIs) and bacterial vaginosis (BV) are associated with increased transmission of HIV, and poor reproductive and sexual health. The burden of STIs/BV among young people is unknown in many high HIV prevalence settings. We conducted an acceptability, feasibility, and prevalence study of home-based sampling for STIs/BV among young men and women aged 15–24 years old in a health and demographic surveillance site (HDSS) in rural KwaZulu-Natal, South Africa.

**Methods and findings:**

A total of 1,342 young people, stratified by age (15–19 and 20–24 years) and sex were selected from the HDSS sampling frame; 1,171/1,342 (87%) individuals had ≥1 attempted home visit between 4 October 2016 and 31 January 2017, of whom 790 (67%) were successfully contacted. Among the 645 who were contacted and eligible, 447 (69%) enrolled. Consenting/assenting participants were interviewed, and blood, self-collected urine (men), and vaginal swabs (women) were tested for herpes simplex virus type 2 (HSV-2), chlamydia, gonorrhoea, syphilis, trichomoniasis, and BV. Both men and women reported that sample collection was easy. Participants disagreed that sampling was painful; more than half of the participants disagreed that they felt anxious or embarrassed. The weighted prevalence of STIs/BV among men and women, respectively, was 5.3% and 11.2% for chlamydia, 1.5% and 1.8% for gonorrhoea, 0% and 0.4% for active syphilis, 0.6% and 4.6% for trichomoniasis, 16.8% and 28.7% for HSV-2, and 42.1% for BV (women only). Of the women with ≥1 curable STI, 75% reported no symptoms. Factors associated with STIs/BV included having older age, being female, and not being in school or working. Among those who participated in the 2016 HIV serosurvey, the prevalence of HIV was 5.6% among men and 19% among women. Feasibility was impacted by the short study duration and the difficulty finding men at home.

**Conclusions:**

A high prevalence of STIs/BV was found in this rural setting with high HIV prevalence in South Africa. Most STIs and HIV infections were asymptomatic and would not have been identified or treated under national syndromic management guidelines. A nested STI/BV survey within a HDSS proved acceptable and feasible. This is a proof of concept for population-based STI surveillance in low- and middle-income countries that could be utilised in the evaluation of STI/HIV prevention and control programmes.

## Abstract 2

Suzanna C Francis and colleagues report on acceptability, feasibility and prevalence results from home-based STI sampling among young men and women in rural KwaZulu-Natal, South Africa.

## Author summary

**Why was this study done?:**

- Adolescents and young adults are particularly vulnerable to sexually transmitted infections (STIs).
- The first strategic direction of the WHO Global Health Sector Strategy on Sexually Transmitted Infections 2016–2021 is to collect information on STI prevalence and incidence across representative populations.
- There is evidence that bacterial vaginosis (BV) is a risk factor for poor birth outcomes and STIs including HIV. The collection of BV prevalence may therefore also be important.
- Developing new cohorts for dedicated STI/BV prevalence studies may not be realistic, particularly in sub-Saharan Africa, where the impact of STIs/BV and their consequences may be greatest.
- Nesting STI/BV surveys within networks of health and demographic surveillance sites (HDSSs) would be an efficient way of providing data to better understand STI epidemiology among adolescents and young people in high HIV prevalence settings.

**What did the researchers do and find?:**

- We carried out a nested STI/BV survey among 1,342 adolescent and young people in an HDSS in KwaZulu-Natal, South Africa, between October 2016 and January 2017.
- Potential participants were contacted at home and invited to participate.
- Participants were interviewed, and samples were collected for STI/BV testing.
- We showed that this study was feasible within the 3.5-month time period: 1,171/1,342 (87%) individuals had ≥1 attempted home visit, of whom 790 (67%) were successfully contacted.
- The study was acceptable: among those contacted and eligible, 447/645 (69%) enrolled. Both men and women reported few problems with sample collection.
- We report a high burden of STIs/BV in this population, particularly of chlamydia (5% in men and 11% in women), herpes simplex virus type 2 (17% in men and 29% in women), and BV (42% in women).

**What do these findings mean?:**

- Nested STI/BV surveys in HDSSs can be feasible and acceptable; however, more survey time is needed to ensure that all potential participants are visited and contacted.
- These studies should be carried out in conjunction with studies to measure STI/BV prevalence in high-risk populations (e.g., female sex workers) to provide robust prevalence estimates.
- These data are essential to advocate, fund, plan, implement, and evaluate interventions for STI prevention and control among adolescents and young people.
- Strategies for the prevention and control of chlamydia, herpes simplex virus type 2, and BV are needed in this population.

## Introduction

In 2012, 286 million people aged 12–24 years lived in Africa, accounting for 18% of the global youth population. By 2040, the number of young people in Africa is projected to increase by 60% to 466 million [1]. Health interventions targeted at this age group are important for current and future adult health and for the health of the next generation. This is particularly true for sexually transmitted infections (STIs), which, when acquired in adolescence, can jeopardise sexual and reproductive health later in life and, for women, the health of their babies. In low- and middle-income countries (LMICs), symptomatic STIs are treated by syndromic management (presumptive treatment for symptomatic people without the use of laboratory tests) [2], but most STIs are asymptomatic and go unnoticed and untreated. Both symptomatic and asymptomatic STIs can cause serious morbidity, including pregnancy complications, cancer, infertility, and enhanced HIV transmission. Many of these sequelae are preventable if STI testing and treatment is implemented. Moreover, there is growing evidence that the common reproductive tract condition bacterial vaginosis (BV) is an independent risk factor for HIV [3,4], and BV-associated microbiota may decrease the efficacy of topical microbicides [5].

High STI prevalence among young people has been observed worldwide and highlights the critical need for global efforts to improve sexual and reproductive health in this population. In an individual participant data meta-analysis of 18 HIV prevention studies among women in sub-Saharan Africa, STI prevalence was higher among young women aged 15–24 years than among older women for all STIs except herpes simplex virus type 2 (HSV-2) [6]; in this age group, the estimated range of prevalence of STIs in South Africa among clinic/community populations was 8.0% to 20.6% for chlamydia, 1.4% to 8.9% for gonorrhoea, 3.1% to 20.0% for trichomoniasis, 31.9% to 53.7% for HSV-2, and 35.8% to 52.4% for BV. In addition, viral STIs such as HSV-2 and human papillomavirus (HPV) infection are often acquired soon after sexual debut, which usually occurs in adolescence, and both are common among young people in sub-Saharan Africa [7–10]. However, many of the studies yielding these results are conducted in urban areas and/or clinical cohorts of adolescents and young adults known to be at high risk of infection. To date, there have been few population estimates of the burden of STIs among adolescent girls and young women and no studies among men [6].

The WHO Global Health Sector Strategy on Sexually Transmitted Infections 2016–2021 has outlined the goals and targets for global STI prevention and control. The first strategic direction is to collect information on STI prevalence and incidence across representative populations [11]. Understanding regional and national STI epidemics is essential to advocate, fund, plan, and implement interventions for STI prevention and control. The strategy also urges LMICs to move from syndromic to aetiologic surveillance of STIs, and to conduct routine surveillance in key populations most at risk for STIs including adolescents. Yet, in resource-limited settings, developing new cohorts for dedicated STI prevalence studies may not be realistic, particularly in sub-Saharan Africa, where the impact of STIs and their consequences may be greatest.

Networks of health and demographic surveillance sites (HDSSs) conducting longitudinal population-based research such as the International Network for the Demographic Evaluation of Populations and their Health (INDEPTH Network) may provide opportunities to obtain representative STI/BV prevalence estimates for adolescents and young people and facilitate community entry and engagement with sensitive topics such as sexual health [12]. However, population-based surveys can be challenging to conduct. Key requirements include the acceptability of being approached at home and home sampling, the feasibility of finding young people at home and a parent available to consent, the receipt of results while maintaining confidentiality, and establishing clinical pathways for the treatment of cases. We conducted a study in the Africa Health Research Institute (AHRI; formerly the Africa Centre for Health and Population Studies) HDSS, a member of the INDEPTH Network, to investigate the acceptability and feasibility of home-based sampling of STIs/BV among young people aged 15–24 years, and to measure prevalence and factors associated with STIs/BV. The background 2011 HIV prevalence in women aged 15–19 years and 20–24 years was 14.7% and 26.5%, respectively, and in men aged 15–19 years and 20–24 years was 7.0% and 10.2%, respectively [13].

## Methods

This study is reported as per the Strengthening the Reporting of Observational Studies in Epidemiology (STROBE) guidelines (S1 STROBE Checklist) [14].

### Setting and sampling

The AHRI HDSS is located in the rural uMkhanyakude district of KwaZulu-Natal, covering an area of 438 km^2^, with a 2016 population of approximately 100,000 people who are members of 12,000 households [15]. Since 2000, annual household-based surveys have been used to collect information on births, deaths, and migration patterns from all household members, including non-residents. In addition, resident household members aged ≥15 years are invited to participate in an annual HIV serosurvey, and to complete a questionnaire on general health and sexual behaviour.

For the STI survey, young men and women who were resident in the HDSS, based on the data collected in the routine household surveillance, and aged 15–24 years as of 19 July 2016 were eligible for inclusion. A random sample of 1,342 young people was selected to obtain a target sample size of 800, allowing for 40% non-contact/refusals. This sample size would have provided acceptable precision for estimating the prevalence of an STI with a prevalence as low as 1.5%. Sampling was stratified by age group (15–19 years and 20–24 years) and sex. The HDSS is divided into 14 subareas; within each stratum, a fixed proportion was sampled from each subarea to reflect the population distribution across the HDSS.

### Ethics, informed consent, and community engagement

The University of KwaZulu-Natal Biomedical Research Ethics Committee, the London School of Hygiene & Tropical Medicine Research Ethics Committee, the Southampton General Hospital Faculty of Medicine Ethics Committee, Hlabisa District Hospital, and the AHRI Somkhele Community Advisory Board approved the study protocol. The STI survey was called *Ukuvikela impilo yetho yokuzalana eyigugu*, isiZulu for ‘protecting our precious reproductive health’. The AHRI Community Engagement Team disseminated information about the study in community dialogues and road shows. Potential participants were contacted at home and invited to participate. Written parental consent was required for participants <18 years old, with participant written assent. Participants aged 18 years or older proved written consent. Participants consented separately for each sample type (vaginal swab [women only], urine [men only], and blood); participants who did not consent for a sample could still enrol in the study. Participants were asked for permission to link their STI survey data with the data collected in the annual routine household and individual surveillance.

### Study procedures

The study team consisted of 2 field workers (1 male and 1 female), 2 female licensed practical nurses, 1 male licensed practical nurse, and 1 male registered nurse team leader, with an intention to match a same-sex nurse to participants whenever possible. The field work was conducted Tuesday to Saturday from 11 AM to 7 PM to maximise the chances of finding participants at home.

After informed consent/assent, the participant had a short computer-assisted personal interview by the study nurse [16]. The interview obtained data on demographics, substance use, sexual behaviour, violence, circumcision (men only), family planning (women only), genital hygiene, and genital symptoms. For questions about sexual behaviour and violence, the participant was asked to self-interview using a tablet device; however, the study nurse was available to support the participant if needed. If a participant reported genital complaints, they were referred to our study nurse in a local primary health clinic for syndromic management as per 2015 South African STI management guidelines [17].

All participants had 8.5 ml of blood drawn for syphilis and HSV-2 testing. For women, the research nurse explained the procedure to self-collect a total of 5 vaginal swabs for testing for chlamydia, gonorrhoea, trichomoniasis, and BV (an additional swab was collected for storage). Swab collection took place in a private setting identified by the participant. Men collected a urine sample for testing for chlamydia, gonorrhoea, and trichomoniasis.

After the sample collection, participants were asked to rate their agreement with 10 statements using a visual analogue scale (VAS) ranging from 0 (easy/agree) to 100 (difficult/disagree) to assess the ease of understanding of consent for the study, the instructions for collecting the sample, and the participant’s experience of participation.

All participants were asked to provide contact information for test results, including their preferred mode of contact for both positive and negative results (e.g., telephone call, SMS message, or WhatsApp message), and ideal hours for contact. We attempted to contact all participants with the results for laboratory-diagnosed curable STIs (chlamydia, gonorrhoea, trichomoniasis, and syphilis). All participants with mobile phones were given 5 South African rand (US$0.37) of air time to contact the study nurses with questions if needed. Participants who had a positive test for a curable STI were referred for free treatment; reimbursement for travel was provided. We traced all cases who were not contactable or did not come to clinic for treatment. We used British Association for Sexual Health and HIV guidelines for the treatment of laboratory-diagnosed chlamydia, gonorrhoea, and trichomoniasis [18–20], and South African STI management guidelines for the treatment of syphilis [17].

### Laboratory methods

Laboratory testing was performed according to manufacturers’ instructions and standard operating procedures in the central AHRI laboratory and Global Clinical and Viral Laboratory in Durban, South Africa. Serum samples were used to test for IgG antibodies for HSV-2 by a type-specific ELISA (Kalon Biological, Guildford, UK). Syphilis infection was determined by the Determine Syphilis TP rapid test (Alere, Waltham, MA, US) in the central AHRI laboratory. All positives were confirmed at the Global Clinical and Viral Laboratory with *Treponema pallidum* haemagglutination (TPHA) (Randox Laboratories, Crumlin, UK) and tested with the Venereal Disease Research Laboratory (VDRL) test (Omega Diagnostics, Alva, UK) using a reverse algorithm as per South African STI management guidelines [17] due to the young age of participants (i.e., unlikely to have treated past infections). Syphilis infection was defined as follows: negative, TPHA−/VDRL−; early or previously treated infection, TPHA+/VDRL−; and active syphilis, TPHA+/VDRL+ low titre [<1:8] or TPHA+/VDRL+ high titre [≥1:8].

Vaginal swabs were used to prepare a slide at the home and air dried. Slides were transported to the central AHRI laboratory, methanol-affixed, Gram stained, and examined for BV using the Nugent score [21]. A Nugent score of 0–3 indicated normal microbiota, 4–6 indicated intermediate microbiota, and 7–10 indicated BV. Vaginal swabs (women) and urine (men) were sent to Global Clinical and Viral Laboratory for testing by real-time PCR for *Neisseria gonorrhoeae*, *Chlamydia trachomatis*, and *Trichomonas vaginalis*. Detection was carried out using the Lightmix Kit *Neisseria gonorrhoeae*, the Lightmix Kit *Chlamydia trachomatis*, and the Lightmix Kit *Trichomonas vaginalis* (TIB MOLBIOL, Berlin, Germany) following the manufacturer’s instructions. All positive tests for *N*. *gonorrhoeae* were confirmed using GeneXpert (Cepheid, Sunnyvale, CA, US). The confirmation test should have a higher specificity than the first test; the GeneXpert *N*. *gonorrhoeae* detection probe has 2 primer sets that increase the specificity needed for the *N*. *gonorrhoeae* confirmation [22,23]. External quality controls were carried out quarterly for real-time PCR with the College of American Pathologists.

### Data management and statistical methods

Data were captured electronically using REDCap software [24]. Range and consistency checks were done automatically during data capture; further data cleaning and analysis was done using Stata 14 (College Station, TX, US). All questions required a response to minimise missing data, although participants could reply ‘don’t know’ or ‘prefer not to say’.

The statistical analysis plan was prepared prior to the statistical analysis (S1 Analysis). Changes in response to peer review of this paper included the inclusion of other STIs in the BV risk factor analysis, and the inclusion of transactional sex in each risk factor analysis. Continuous variables were summarised using means and standard deviations or medians and interquartile ranges; categorical data were summarised using frequency counts and percentages. Missing data were not imputed.

The acceptability and feasibility of our survey were measured by the following outcomes: proportion of participants who were selected and contactable, the proportion of those contacted who agreed to participate, the proportion who agreed to each sample collection (e.g., blood, vaginal swabs, and urine), median and interquartile range of responses to a VAS measuring acceptability post-sampling, and proportion of cases who presented for treatment. We also estimated STI/BV prevalence and explored factors associated with any curable STI (chlamydia, gonorrhoea, syphilis, and trichomoniasis), HSV-2, and BV.

The number of individuals who were successfully contacted and who consented to participate were tabulated by sex, age group, residence location (urban/peri-urban/rural), household socioeconomic status, education level, and HIV status using linked data from the HDSS. Characteristics of individuals who participated and the remainder in the eligibility list were compared using chi-squared tests.

The prevalence estimate of each STI or BV, and its 95% confidence interval, was calculated overall and by sex; prevalence estimates were weighted to account for the stratified sample design and non-response, calculated as the inverse probability of study participation in strata defined by age group, sex, and residence location (urban/peri-urban/rural). We compared these results to unweighted prevalence and prevalence weighted for the stratified sample design only.

Logistic regression was used to estimate odds ratios and 95% CIs for factors associated with the presence of any curable STI (chlamydia, gonorrhoea, syphilis, or trichomoniasis), of HSV-2, and of BV; separate models were developed for each outcome. Potential factors associated with curable STIs, HSV-2, and BV were examined using a conceptual framework with 3 levels: sociodemographic factors, modifiable behavioural factors (including genital hygiene), and sexual behaviour and violence. For each outcome, age and sex (except for BV, which was in women only) were considered a priori confounders and were included in all models. Sociodemographic factors whose age- and sex-adjusted associations with the outcome were significant at *P <* 0.10 were included in a multivariable model; those remaining associated at *P <* 0.10 were retained in a core model. Behavioural factors were then added to this core model one by one; those that were associated with the outcome at *P <* 0.10, after adjusting for sociodemographic factors, were included in a multivariable model and retained if they remained associated at *P <* 0.10. Associations with sexual behavioural and violence factors were subsequently determined in a similar way. Many of the questions about sexual relationships were asked only if participants reported having ever had sex, so analyses of these variables were restricted to that subgroup.

## Results

### Acceptability and feasibility

**Fig 1:**
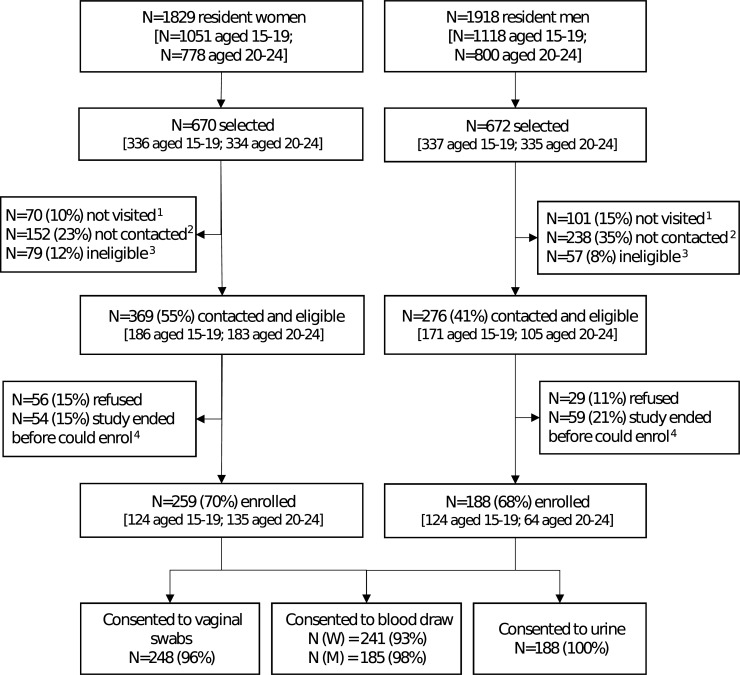
Flow diagram for enrolment in a population-based sexually transmitted infection survey among young people aged 15–24 years in rural Kwa-Zulu Natal. ^1^Not visited: no visit was made to the household because the study ended. ^2^Not contacted: at least 1 visit was made to the household, but either a parent (if selected individual <18 years) or the individual was unavailable. ^3^Ineligible: individuals who were found to have out-migrated from the health and demographic surveillance site (*N =* 73 women and 53 men) or who were not capable of providing consent (*N =* 6 women and 4 men). ^4^Individuals who were interested in the study, but could not enrol because study ended.

The field work took place from 4 October 2016 to 31 January 2017. Due to unexpected time limitations, only 1 visit attempt per selected individual was carried out from November to January to attempt coverage in subareas (a total of 14 subareas), but not all selected young people were visited. Among the 1,342 individuals selected, 1,171 (87%) had ≥1 attempted home visit, of whom 781 (67%) were successfully contacted (Fig 1). Of those who were contacted, 645 (83%) were still eligible. Among those contacted and eligible, 447 (69%) enrolled. Individuals aged 20–24 years were less likely to be contacted than those aged 15–19 years (63% versus 70% of those with an attempted visit, *P =* 0.01) and less likely to be eligible after contact was made (mostly due to migration). Men were less likely to be contacted than women (58% versus 75%, *P <* 0.001). Overall, there was strong evidence that individuals who were sampled but did not enrol were more likely to be older, male, and from rural or urban areas, and to have completed secondary education or above, compared with those who enrolled (S1 Table).

**Fig 2:**
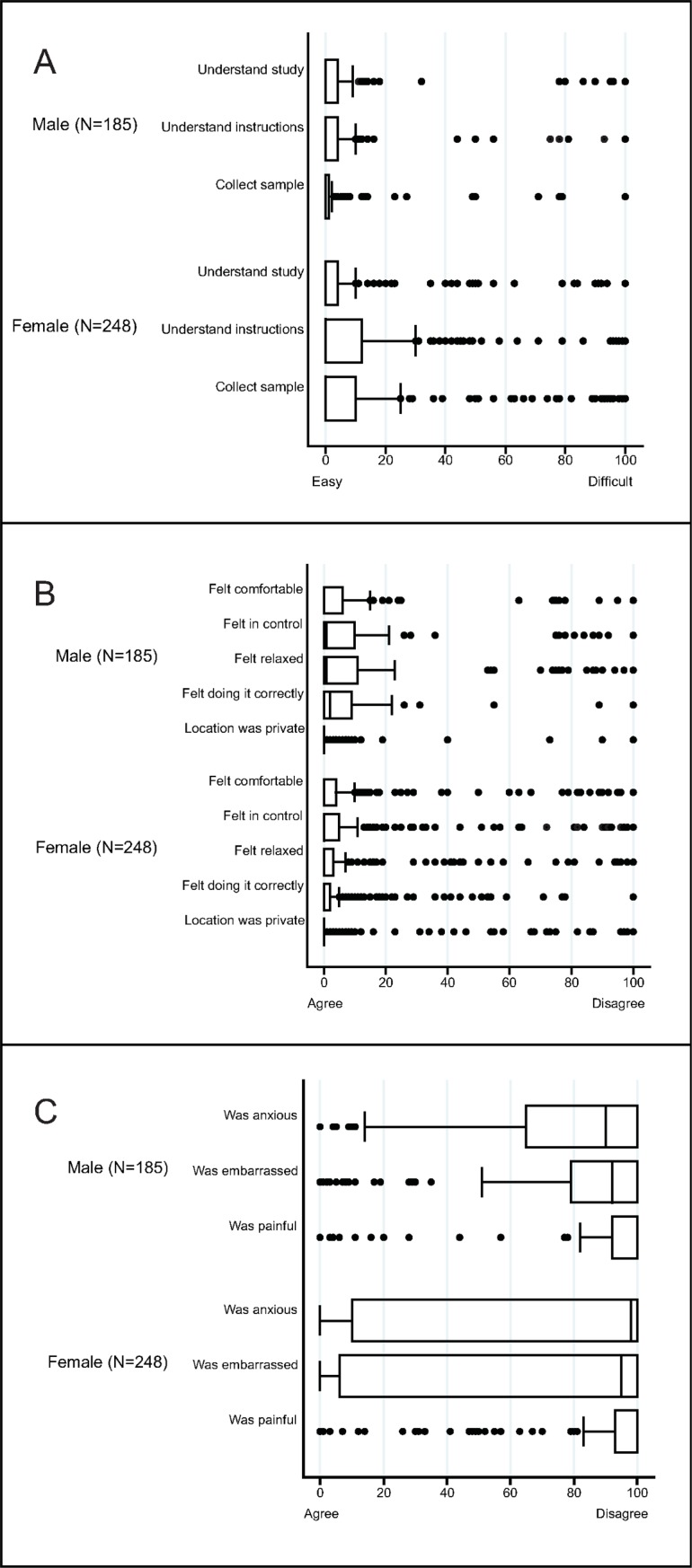
Box and whisker plots of the acceptability of sampling in a population-based sexually transmitted infection/bacterial vaginosis survey among young people aged 15–24 years in rural KwaZulu-Natal. The vertical line within the box indicates the median, the boundaries of the box indicate the interquartile range (25th and 75th percentiles), and the whiskers indicate values that are within 1.5 times the interquartile range above the 75th percentile, or 1.5 times the interquartile range below the 25th percentile. Values outside that range are plotted as individual points, e.g., the medians for (A) equal 0. (A) Ease of understanding the study and instructions, and the ease of sample collection. (B) Experience of self-collecting urine (males) or vaginal swabs (women)—positive items. (C) Experience of self-collecting urine (males) or vaginal swabs (women)—negative items.

Of those enrolled, 96% of women provided all vaginal swabs and 93% provided blood samples; all men provided urine samples and 98% provided blood samples. Both men and women reported that it was easy to understand how to collect urine and vaginal swabs, respectively (Fig 2A). Participants agreed they felt comfortable, in control, relaxed, and confident of their ability to collect the sample correctly (Fig 2B). Participants disagreed that sampling was painful. Most men disagreed that they felt anxious or embarrassed, and over half of women disagreed that they were anxious or embarrassed (Fig 2C).

Of those who provided samples, 206/245 (84%) of individuals aged 15–19 years and 184/192 (96%) of individuals aged 20–24 years enrolled had access to a telephone to receive results. Of those, the majority preferred a telephone call for both positive and negative results (59% and 57%, respectively), followed by an SMS message (37% and 39%, respectively). Few chose to receive their results (positive or negative) by WhatsApp message (4% and 4%, respectively). These results were similar by sex and age, although a higher proportion of males than females preferred to receive their results by telephone (S2 Table).

Fifty-five participants had ≥1 curable STI and were invited to the clinic for management: 52 (95%) came on their own, and 3 had to be traced.

### Results of behavioural questionnaire

Most participants were currently enrolled in school (Table 1). Few participants were working (11% of men and 5% of women). Proportionally, more men aged 20–24 years reported having ever smoked a cigarette compared to women of the same age (17% versus 8%, respectively). Conversely, proportionally fewer men compared to women reported having ever had at least 1 drink of alcohol (23% versus 51%, respectively). A small proportion of participants reported cannabis use: 8% among men aged 20–24 years and 4% among women of the same age. Few participants reported using other drugs (1%).

**Table 1:** Baseline characteristics of participants in a population-based sexually transmitted infection survey among young people aged 15–24 years in rural KwaZulu-Natal (*N* = 447).

Characteristic^1^	Male			Female			Overall		
	15–19 years (*N =* 124)	20–24 years (*N =* 64)	Total (*N =* 188)	15–19 years (*N =* 124)	20–24 years (*N =* 135)	Total (*N =* 259)	15–19 years (*N =* 248)	20–24 years (*N =* 199)	Total (*N =* 447)
**Highest level of education**									
Primary	16 (12.9%)	3 (4.7%)	19 (10.1%)	5 (4.0%)	9 (6.7%)	14 (5.4%)	21 (8.5%)	12 (6.0%)	33 (7.4%)
Secondary	90 (72.6%)	21 (32.8%)	111 (59.0%)	106 (85.5%)	52 (38.5%)	158 (61.0%)	196 (79.0%)	73 (36.7%)	269 (60.2%)
Matriculation or above^2^	18 (14.5%)	40 (62.5%)	58 (30.9%)	13 (10.5%)	74 (54.8%)	87 (33.6%)	31 (12.5%)	114 (57.3%)	145 (32.4%)
**Socioeconomic status**^**3**^									
Low	53 (42.7%)	27 (42.2%)	80 (42.6%)	57 (46.3%)	60 (44.8%)	117 (45.5%)	110 (44.5%)	87 (43.9%)	197 (44.3%)
Middle	30 (24.2%)	18 (28.1%)	48 (25.5%)	24 (19.5%)	38 (28.4%)	62 (24.1%)	54 (21.9%)	56 (28.3%)	110 (24.7%)
High	41 (33.1%)	19 (29.7%)	60 (31.9%)	42 (34.2%)	36 (26.9%)	78 (30.4%)	83 (33.6%0	55 (27.8%)	138 (31.0%)
**Currently in school**	114 (91.9%)	30 (46.9%)	144 (76.6%)	112 (90.3%)	47 (35.1%)	159 (61.6%)	226 (91.1%)	77 (38.9%)	303 (67.9%)
**Working**	12 (9.8%)	8 (12.5%)	20 (10.7%)	7 (5.6%)	7 (5.2%)	14 (5.4%)	19 (7.7%)	15 (7.5%)	34 (7.6%)
**Ever smoked a cigarette**	3 (2.5%)	11 (17.2%)	14 (7.5%)	13 (10.6%)	11 (8.1%)	24 (9.3%)	16 (6.5%)	22 (11.1%)	38 (8.6%)
**Ever used cannabis**	1 (0.8%)	5 (7.8%)	6 (3.2%)	8 (6.5%)	5 (3.7%)	13 (5.0%)	9 (3.6%)	10 (5.0%)	19 (4.3%)
**Ever used other drugs**	2 (1.6%)	—	2 (1.1%)	1 (0.8%)	2 (1.5%)	3 (1.2%)	3 (1.2%)	2 (1.0%)	5 (1.1%)
**Ever drank 1 drink of alcohol**	26 (21.7%)	17 (26.6%)	43 (23.4%)	57 (47.1%)	73 (54.5%)	130 (51.0%)	83 (34.4%)	90 (45.5%)	173 (39.4%)
**Ever been circumcised**	71 (57.3%)	25 (39.1%)	96 (51.1%)	—	—	—	—	—	—
**Ever cleansed inside vagina**	—	—	—	13 (10.7%)	28 (20.9%)	41 (16.1%)	—	—	—
**Ever had sex**	29 (23.6%)	32 (52.5%)	61 (33.2%)	54 (46.2%)	101 (77.7%)	155 (62.8%)	83 (34.6%)	133 (69.6%)	216 (50.1%)
**Current use of any contraception**	—	—	—	27 (22.5%)	67 (50.4%)	94 (37.2%)	—	—	—
**Condom at last sex**	16 (55.2%)	20 (60.6%)	36 (58.1%)	26 (49.1%)	36 (36.4%)	62 (40.8%)	42 (51.2%)	56 (42.4%)	98 (45.8%)
**Last partner discussed his/her HIV status**	8 (27.6%)	13 (44.8%)	21 (36.2%)	25 (46.3%)	60 (61.2%)	85 (55.9%)	33 (39.8%)	73 (57.5%)	106 (50.5%)
**Knew last partner’s HIV status**	9 (31.0%)	13 (43.3%)	22 (37.3%)	28 (49.1%)	59 (59.0%)	87 (55.4%)	37 (43.0%)	72 (55.4%)	109 (50.5%)
**Discussed your HIV status with last partner**	12 (50.0%)	14 (46.7%)	26 (48.1%)	28 (53.8%)	62 (68.9%)	90 (63.4%)	40 (52.6%)	76 (63.3%)	116 (59.2%)
**Number of current sexual partners**									
None	94 (78.3%)	29 (49.2%)	123 (68.7%)	63 (57.3%)	29 (23.2%)	92 (39.1%)	157 (68.3%)	58 (31.5%)	215 (51.9%)
1	21 (17.5%)	21 (35.6%)	42 (23.5%)	44 (40.0%)	91 (72.8%)	135 (57.4%)	65 (28.3%)	112 (60.9%)	177 (42.8%)
2 or more	5 (4.2%)	9 (15.3%)	14 (7.8%)	3 (2.7%)	5 (4.0%)	8 (3.4%)	8 (3.5%)	14 (7.6%)	22 (5.3%)
**Oral sex, received**	12 (9.7%)	8 (12.9%)	20 (10.8%)	21 (17.5%)	59 (46.1%)	80 (32.3%)	33 (13.5%)	67 (35.3%)	100 (23.0%)
**Oral sex, provided**	9 (7.3%)	7 (11.5%)	16 (8.7%)	11 (9.2%)	38 (30.2%)	49 (19.9%)	20 (8.2%)	45 (24.1%)	65 (15.1%)
**Ever had anal sex**	2 (1.6%)	—	2 (1.1%)	6 (5.0%)	6 (4.7%)	12 (4.8%)	8 (3.3%)	6 (3.2%)	14 (3.2%)
**Violence, perpetrator**	5 (4.1%)	3 (4.9%)	8 (4.4%)	3 (2.6%)	8 (6.2%)	11 (4.3%)	8 (3.4%)	11 (5.8%)	19 (4.4%)
**Violence, victim**	8 (6.5%)	3 (4.8%)	11 (5.9%)	7 (5.8%)	19 (15.1%)	26 (10.5%)	15 (6.1%)	22 (11.7%)	37 98.7%)
**HIV status**^**4**^									
Positive	2 (3.6%)	5 (19.2%)	7 (8.5%)	6 (8.6%)	23 (29.5%)	29 (19.6%)	8 (6.4%)	28 (26.9%)	36 (15.7%)
Negative	54 (96.4%)	21 (80.8%)	75 (91.5%)	64 (91.4%)	55 (70.5%)	119 (80.4%)	118 (93.6%)	76 (73.1%)	194 (84.3%)

Data given as *N* (percent).

^1^Proportions of individuals who responded to study questions (excluding those who preferred not to answer).

^2^Matriculation is the qualification received upon graduating from high school, and is the minimum requirement for entrance to university.

^3^Socioeconomic status from a household-level asset index constructed based on ownership of 27 common household items and housing construction, using principal component analysis. Derived from linking the annual health and demographic surveillance site household-level survey in 2015/2016.

^4^Linked to HIV test result among individuals who participated in the 2016 serosurvey.

In all, 51% of men reported circumcision: younger men were more likely to be circumcised (Table 1). Sixteen percent of women reported using intravaginal cleansing; older women were more likely to report intravaginal cleansing. In all, 23% of women aged 15–19 years and 50% of women aged 20–24 years reported use of any type of contraception.

In all, 61 (33%) men and 155 (63%) women reported having had sexual intercourse; of these, the median (IQR) number of lifetime partners was 4 (2–6) for men and 3 (2–3) for women. A larger proportion of men than women reported having used a condom at last intercourse (58% versus 41%, respectively). A smaller proportion of men than women reported knowing their last partner’s HIV status (37% versus 55%, respectively), and a smaller proportion of men than women reported discussing their own HIV status with their last partner (48% of men versus 63% of women).

More women aged 20–24 years reported providing (32%) or receiving oral sex (46%) than men of the same age group (12% and 13%, respectively). Few participants reported ever having anal sex, and fewer men than women (1% versus 5%). Among men who participated in the 2016 HIV serosurvey, the prevalence of laboratory-diagnosed HIV among those aged 15–19 years was 4% and among those aged 20–24 years was 19%. Among women, the prevalence of laboratory-diagnosed HIV among those aged 15–19 years was 9% and among those aged 20–24 years was 30%.

### Prevalence of STIs/BV

Weighted prevalence from Table 2 shows a high prevalence of chlamydia in men aged 20–24 years (12.6%; 95% CI 6.4%–23.3%) and women in both age groups (15–19 years: 11.7%; 95% CI 6.8%–19.3%; 20–24 years: 10.2%; 95% CI 6.0%–16.9%). The prevalence of gonorrhoea was low, from 0 cases among men aged 20–24 years to 3.2% (95% CI 1.2%–8.2%) in women of the same age group. There was 1 case of active syphilis—the overall prevalence of active syphilis was 0.1%. There were 5 TPHA−/VDRL+ samples. The prevalence of trichomoniasis was lower in men compared with women (0.6% [95% CI 0.1%–4.0%] versus 4.6% [95% CI 2.6%–7.9%]); the highest prevalence was among women aged 20–24 years. In all, 14% of individuals had a curable STI (chlamydia, gonorrhoea, syphilis, or trichomoniasis). Of these, 75% reported no symptoms. The prevalence of HSV-2 was lower in men compared with women (16.8% [95% CI 11.3%–24.1%] versus 28.7% [95% CI 23.3%–34.7%]), with the highest prevalence among women aged 20–24 years. The prevalence of BV was 41.1% (95% CI 32.3%–50.5%) among women aged 15–19 years and 44.2% (95% CI 35.5%–53.2%) among women aged 20–24 years. Prevalence weighted for sampling and non-response (Table 2) was similar to unweighted prevalence and prevalence using sampling weights only (S3 Table).

**Table 2:** Prevalence of STIs weighted for sampling and non-response in a population-based STI survey among young people aged 15–24 years in rural KwaZulu-Natal (*N =* 447).

STI	Male			Female			All participants		
	15–19 years (*N =* 124)	20–24 years (*N =* 64)	All males (*N =* 188)	15–19 years (*N =* 124)	20–24 years (*N =* 135)	All females (*N =* 259)	15–19 years (*N =* 248)	20–24 years (*N =* 199)	All participants (*N =* 477)
**Chlamydia**									
Positive	1.6% (0.4–6.1)	12.6% (6.4–23.3)	5.3% (3.0–9.4)	11.7% (6.8–19.3)	10.2% (6.0–16.9)	11.2% (7.5–16.4)	6.2% (3.8–10.1)	11.5% (7.4–17.5)	8.1% (5.8–11.1)
**Gonorrhoea**									
Positive	2.3% (0.7–7.1)	0	1.5% (0.5–4.7)	1.1% (0.3–4.5)	3.2% (1.2–8.2)	1.8% (0.8–4.1)	1.8% (0.7–4.3)	1.5% (0.6–3.9)	1.7% (0.8–3.3)
**Syphilis**									
Active	0	0	0	0	0.8% (0.1–5.8)	0.3% (0.0–2.1)	0	0.4% (0.1–2.7)	0.1% (0.0–0.9)
Early/previously treated	0	1.2% (0.2–8.5)	0.4% (0.1–3.0)	0	3.3% (1.2–8.4)	1.1% (0.4–3.0)	0	2.2% (0.9–5.3)	0.8% (0.3–1.8)
**Trichomoniasis**									
Positive	0	1.7% (0.2–11.1)	0.6% (0.1–4.0)	2.0% (0.5–7.7)	9.4% (5.4–16.0)	4.6% (2.6–7.9)	0.9% (0.2–3.6)	5.3% (3.0–9.2)	2.4% (1.4–4.2)
**HSV-2**									
Positive	12.1% (7.3–19.2)	25.8% (14.3–41.9)	16.8% (11.3–24.1)	18.1% (11.8–26.8)	48.0% (39.2–56.9)	28.7% (23.3–34.7)	14.8% (10.7–20.1)	36.1% (28.4–44.6)	22.2% (18.2–26.8)
**Bacterial vaginosis**									
Intermediate	—	—	—	9.9% (5.5–17.2)	11.3% (6.8–18.3)	10.4% (6.8–15.4)	—	—	—
Positive	—	—	—	41.1% (32.3–50.5)	44.2% (35.5–53.2)	42.1% (35.5–49.0)	—	—	—

Data given as percent (95% CI).

HSV-2, herpes simplex virus type 2; STI, sexually transmitted infection.

### Factors associated with STIs/BV

In the adjusted analysis of factors associated with curable STIs (chlamydia, gonorrhoea, syphilis, and trichomoniasis), participants aged 20–24 years and women had more than twice the odds of having a curable STI compared to participants aged 15–19 years and men, respectively (Table 3). Having a higher number of lifetime sexual partners was associated with having a curable STI (*P =* 0.038). Reporting having had sexual intercourse was strongly associated with having a curable STI.

**Table 3:** Factors associated with chlamydia, gonorrhoea, syphilis and trichomoniasis in a population-based STI survey among young people aged 15–24 years in rural KwaZulu-Natal (*N =* 447).

Factor	Number with any curable STI/*N* (%)	Crude OR (95% CI); *P* value	Age- and sex-adjusted OR (95% CI); *P* value	Adjusted OR^1^ (95% CI); *P* value
***Sociodemographics***				
**Age group**		*P <* 0.001	*P =* 0.001	*P =* 0.001
15–19 years	20/245 (8.2%)	1	1.00 (1.00–1.00)	1
20–24 years	40/191 (20.9%)	2.98 (1.68–5.30)	2.64 (1.47–4.73)	2.64 (1.47–4.73)
**Sex**		*P =* 0.001	*P =* 0.006	*P =* 0.006
Male	14/188 (7.4%)	1	1	1
Female	46/248 (18.5%)	2.83 (1.50–5.32)	2.45 (1.29–4.65)	2.45 (1.29–4.65)
**In school or working**		*P =* 0.001	*P =* 0.268	*P =* 0.268
No	29/128 (22.7%)	1	1	1
Yes	31/308 (10.1%)	0.38 (0.22–0.67)	0.69 (0.35–1.33)	0.69 (0.35–1.33)
**Socioeconomic status**^**2**^		*P =* 0.276	*P =* 0.287	*P =* 0.287
Low	32/192 (16.7%)	1	1	1
Middle	14/109 (12.8%)	0.74 (0.37–1.45)	0.68 (0.34–1.36)	0.68 (0.34–1.36)
High	14/133 (10.5%)	0.59 (0.30–1.15)	0.61 (0.30–1.21)	0.61 (0.30–1.21)
**Highest level of education completed**		*P =* 0.485	*P =* 0.774	*P =* 0.774
Primary	5/33 (15.2%)	1	1	1
Secondary	32/263 (12.2%)	0.78 (0.28–2.15)	0.79 (0.27–2.31)	0.79 (0.27–2.31)
Matriculation or above^3^	23/140 (16.4%)	1.10 (0.38–3.15)	0.68 (0.22–2.07)	0.68 (0.22–2.07)
***Behaviour***				
**Ever smoked a cigarette**		*P =* 0.718	*P =* 0.915	*P =* 0.827
No	54/395 (13.7%)	1	1	1
Yes	6/38 (15.8%)	1.18 (0.47–2.97)	1.05 (0.41–2.68)	0.90 (0.34–2.35)
**Ever drank alcohol**		*P =* 0.014	*P =* 0.150	*P =* 0.150
No	27/259 (10.4%)	1	1	1
Yes	32/169 (18.9%)	2.01 (1.15–3.49)	1.54 (0.86–2.75)	1.54 (0.86–2.75)
***Sexual behaviour and violence***				
**Genital touching**		*P =* 0.001	*P =* 0.044	*P =* 0.296
No	24/258 (9.3%)	1	1	1
Yes	36/178 (20.2%)	2.47 (1.42–4.31)	1.82 (1.02–3.26)	1.48 (0.71–3.09)
**Oral sex, received**		*P =* 0.106	*P =* 0.963	*P =* 0.201
No	39/326 (12.0%)	1	1	1
Yes	18/98 (18.4%)	1.66 (0.90–3.05)	0.98 (0.51–1.91)	0.62 (0.30–1.29)
**Oral sex, provided**		*P =* 0.156	*P =* 0.850	*P =* 0.626
No	45/357 (12.6%)	1	1	1
Yes	12/62 (19.4%)	1.66 (0.82–3.36)	1.07 (0.51–2.26)	0.83 (0.38–1.79)
**Ever had sex**		*P <* 0.001	*P =* 0.004	*P =* 0.009
No	13/210 (6.2%)	1	1	1
Yes	45/210 (21.4%)	4.13 (2.16–7.92)	2.77 (1.38–5.55)	2.67 (1.28–5.55)
**Number of lifetime sexual partners**		*P <* 0.001	*P =* 0.014	*P =* 0.038
None	13/210 (6.2%)	1	1	1
1	17/82 (20.7%)	3.96 (1.83–8.60)	2.81 (1.25–6.33)	2.78 (1.19–6.50)
2 or more	23/104 (22.1%)	4.30 (2.08–8.91)	2.88 (1.32–6.28)	2.47 (1.07–5.70)
**Violence, perpetrator**		*P =* 0.681	*P =* 0.891	*P =* 0.395
No	53/399 (13.3%)	1	1	1
Yes	3/18 (16.7%)	1.31 (0.37–4.66)	1.10 (0.29–4.09)	0.49 (0.09–2.55)
**Violence, victim**		*P =* 0.006	*P =* 0.040	*P =* 0.126
No	45/387 (11.6%)	1	1	1
Yes	10/35 (28.6%)	3.04 (1.37–6.74)	2.39 (1.04–5.49)	1.96 (0.83–4.65)

^1^Sociodemographic variables adjusted for age and sex. Behavioural variables adjusted for age, sex, and ever drank alcohol. Sexual behaviour and violence variables adjusted for age, sex, ever drank alcohol, ever had sex, and having been a violence victim. Number of lifetime sexual partners was not included for the adjustment with sexual behaviour due to collinearity with ever had sex. Excludes those who preferred not to answer.

^2^Socioeconomic status from a household-level asset index constructed based on ownership of 27 common household items and housing construction, using principal component analysis. Derived from linking the annual health and demographic surveillance site household-level survey in 2015/2016.

^3^Matriculation is the qualification received upon graduating from high school, and is the minimum requirement for entrance to university.

OR, odds ratio; STI, sexually transmitted infection.

In the adjusted analysis of factors associated with HSV-2, participants aged 20–24 years and women had twice the odds of HSV-2 infection compared to participants aged 15–19 years and men, respectively (Table 4). Participants currently enrolled in school or working had less than half the odds of HSV-2 infection compared to those who were neither in school nor working.

**Table 4:** Factors associated with HSV-2 in a population-based sexually transmitted infection survey among young people aged 15–24 years in rural Kwa-Zulu Natal (*N =* 419^1^).

Factor	Number with HSV-2/*N* (%)	Crude OR (95% CI); *P* value	Age- and sex-adjusted OR (95% CI); *P* value	Adjusted OR^2^ (95% CI); *P* value
***Sociodemographics***				
**Age group**		*P <* 0.001	*P <* 0.001	*P =* 0.004
15–19 years	35/233 (15.0%)	1	1	1
20–24 years	73/186 (39.2%)	3.65 (2.30–5.82)	3.29 (2.05–5.28)	2.23 (1.29–3.83)
**Sex**		*P <* 0.001	*P =* 0.001	*P =* 0.006
Male	29/183 (15.8%)	1	1	1
Female	79/236 (33.5%)	2.67 (1.65–4.32)	2.28 (1.39–3.74)	2.03 (1.22–3.37)
**In school or working**		*P <* 0.001	*P =* 0.003	*P =* 0.003
No	57/125 (45.6%)	1	1	1
Yes	51/294 (17.3%)	0.25 (0.16–0.40)	0.44 (0.25–0.75)	0.44 (0.25–0.75)
**Socioeconomic status**^**3**^		*P =* 0.437	*P =* 0.550	*P =* 0.691
Low	51/184 (27.7%)	1	1	1
Middle	29/105 (27.6%)	1.00 (0.58–1.70)	0.92 (0.52–1.62)	0.85 (0.47–1.52)
High	28/129 (21.7%)	0.72 (0.43–1.23)	0.73 (0.42–1.28)	0.79 (0.45–1.39)
**Highest level of education completed**		*P =* 0.007	*P =* 0.413	*P =* 0.743
Primary	9/31 (29.0%)	1	1	1
Secondary	51/251 (20.3%)	0.62 (0.27–1.44)	0.59 (0.24–1.45)	0.72 (0.28–1.80)
Matricuation or above^4^	48/137 (35.0%)	1.32 (0.56–3.09)	0.76 (0.30–1.93)	0.70 (0.27–1.80)
***Behaviour***				
**Ever smoked a cigarette**		*P =* 0.289	*P =* 0.488	*P =* 0.400
No	96/381 (25.2%)	1	1	1
Yes	12/36 (33.3%)	1.48 (0.71–3.08)	1.31 (0.61–2.80)	1.40 (0.64–3.04)
**Ever drank alcohol**		*P =* 0.009	*P =* 0.244	*P =* 0.204
No	52/250 (20.8%)	1	1	1
Yes	52/161 (32.3%)	1.82 (1.16–2.85)	1.34 (0.82–2.18)	1.38 (0.84–2.27)
***Sexual behaviour and violence***				
**Genital touching**		*P <* 0.001	*P =* 0.001	*P =* 0.255
No	42/247 (17.0%)	1	1	1
Yes	66/172 (38.4%)	3.04 (1.93–4.78)	2.24 (1.39–3.61)	1.40 (0.78–2.52)
**Oral sex, received**		*P =* 0.008	*P =* 0.741	*P =* 0.503
No	73/314 (23.2%)	1	1	1
Yes	35/94 (37.2%)	1.96 (1.20–3.21)	1.10 (0.63–1.90)	0.82 (0.46–1.47)
**Oral sex, provided**		*P =* 0.022	*P =* 0.577	*P =* 0.859
No	81/343 (23.6%)	1	1	1
Yes	23/61 (37.7%)	1.96 (1.10–3.48)	1.19 (0.64–2.22)	0.94 (0.49–1.81)
**Ever had sex**		*P <* 0.001	*P =* 0.004	*P =* 0.012
No	31/204 (15.2%)	1	1	1
Yes	76/201 (37.8%)	3.39 (2.11–5.47)	2.14 (1.27–3.58)	1.96 (1.16–3.32)
**Number of lifetime sexual partners**		*P <* 0.001	*P =* 0.035	*P =* 0.085
None	31/204 (15.2%)	1	1	1
1	21/78 (26.9%)	2.06 (1.10–3.86)	1.40 (0.72–2.73)	1.29 (0.65–2.56)
2 or more	39/100 (39.0%)	3.57 (2.05–6.21)	2.22 (1.21–4.06)	2.01 (1.08–3.73)
**Violence, perpetrator**		*P =* 0.791	*P =* 0.558	*P =* 0.394
No	102/386 (26.4%)	1	1	1
Yes	4/17 (23.5%)	0.86 (0.27–2.69)	0.70 (0.21–2.33)	0.59 (0.18–1.98)
**Violence, victim**		*P =* 0.102	*P =* 0.400	*P =* 0.532
No	94/373 (25.2%)	1	1	1
Yes	13/34 (38.2%)	1.84 (0.89–3.81)	1.40 (0.64–3.08)	1.29 (0.58–2.83)

^1^Number less than 447 due to the number of samples collected for testing for HSV-2.

^2^Sociodemographic variables adjusted for age, sex, and in school/working. Behaviour variables adjusted for age, sex, and in school/working. Sexual behaviour and violence variables adjusted for age, sex, in school/working, and ever had sex. Number of lifetime sexual partners was not included for the adjustment with sexual behaviour due to collinearity with ever had sex. Excludes those who preferred not to answer.

^3^Socioeconomic status from a household-level asset index constructed based on ownership of 27 common household items and housing construction, using principal component analysis. Derived from linking the annual health and demographic surveillance site household-level survey in 2015/2016.

^4^Matriculation is the qualification received upon graduating from high school, and is the minimum requirement for entrance to university.

HSV-2, herpes simplex virus type 2; OR, odds ratio.

In the adjusted analysis of factors associated with BV, there was weak evidence that being currently enrolled in school or working was associated with a diagnosis of BV (Table 5). Those having ever drunk alcohol had twice the odds of a diagnosis of BV, and there was weak evidence that having ever smoked a cigarette was associated with a diagnosis of BV. Independently, those reporting genital touching and having ever had sex had twice the odds of a diagnosis of BV. Participants who were HSV-2 seropositive had 4 times the odds of a diagnosis of BV.

**Table 5:** Factors associated with BV in young women in a population-based STI survey among young people aged 15–24 years in rural Kwa-Zulu Natal (*N =* 239^1^).

Factor	Number with BV/*N* (%)	Crude OR (95% CI); *P* value	Age-adjusted OR (95% CI); *P* value	Adjusted OR^2^ (95% CI); *P* value
***Sociodemographics***				
**Age group**		*P =* 0.640	*P =* 0.640	*P =* 0.469
15–19 years	49/119 (41.2%)	1	1	1
20–24 years	53/120 (44.2%)	1.13 (0.68–1.89)	1.13 (0.68–1.89)	0.79 (0.42–1.49)
**In school or working**		*P =* 0.059	*P =* 0.050	*P =* 0.050
No	45/89 (50.6%)	1	1	1
Yes	57/150 (38.0%)	0.60 (0.35–1.02)	0.52 (0.27–1.00)	0.52 (0.27–1.00)
**Socioeconomic status**^**3**^		*P =* 0.092	*P =* 0.090	*P =* 0.128
Low	54/107 (50.5%)	1	1	1
Middle	24/60 (40.0%)	0.65 (0.34–1.24)	0.64 (0.34–1.23)	0.61 (0.31–1.16)
High	24/70 (34.3%)	0.51 (0.27–0.95)	0.51 (0.28–0.96)	0.56 (0.30–1.06)
**Highest level of education completed**		*P =* 0.582	*P =* 0.643	*P =* 0.816
Primary	7/13 (53.8%)	1	1	1
Secondary	60/148 (40.5%)	0.58 (0.19–1.83)	0.59 (0.19–1.87)	0.73 (0.22–2.39)
Matriculation or above^4^	35/78 (44.9%)	0.70 (0.21–2.27)	0.69 (0.21–2.26)	0.68 (0.20–2.24)
***Behaviour***				
**Ever smoked a cigarette**		*P =* 0.027	*P =* 0.027	*P =* 0.104
No	87/215 (40.5%)	1	1	1
Yes	15/23 (65.2%)	2.76 (1.12–6.79)	2.77 (1.13–6.82)	2.19 (0.85–5.61)
**Ever drank alcohol**		*P =* 0.012	*P =* 0.014	*P =* 0.009
No	39/114 (34.2%)	1	1	1
Yes	61/121 (50.4%)	1.96 (1.16–3.31)	1.94 (1.15–3.29)	2.04 (1.19–3.48)
**Ever cleansed inside vagina**		*P =* 0.569	*P =* 0.545	*P =* 0.861
No	88/200 (44.0%)	1	1	1
Yes	14/36 (38.9%)	0.81 (0.39–1.67)	0.80 (0.38–1.66)	0.93 (0.44–1.99)
**Use hormonal contraception**		*P =* 0.425	*P =* 0.467	*P =* 0.876
No	84/202 (41.6%)	1	1	1
Yes	18/37 (48.6%)	1.33 (0.66–2.69)	1.30 (0.64–2.66)	1.06 (0.49–2.29)
***Sexual behaviour and violence***				
**Genital touching**		*P <* 0.001	*P <* 0.001	*P =* 0.021
No	36/122 (29.5%)	1	1	1
Yes	66/117 (56.4%)	3.09 (1.81–5.27)	3.12 (1.82–5.35)	2.12 (1.12–4.02)
**Oral sex, received**		*P =* 0.014	*P =* 0.013	*P =* 0.682
No	57/158 (36.1%)	1	1	1
Yes	39/73 (53.4%)	2.03 (1.16–3.57)	2.11 (1.17–3.82)	1.16 (0.58–2.31)
**Oral sex, provided**		*P =* 0.068	*P =* 0.077	*P =* 0.936
No	71/186 (38.2%)	1	1	1
Yes	23/43 (53.5%)	1.86 (0.95–3.63)	1.86 (0.94–3.71)	0.97 (0.45–2.11)
**Ever had sex**		*P <* 0.001	*P <* 0.001	*P =* 0.031
No	22/85 (25.9%)	1	1	1
Yes	74/143 (51.7%)	3.07 (1.71–5.52)	3.43 (1.85–6.38)	2.14 (1.07–4.27)
**Number of lifetime sexual partners**		*P =* 0.003	*P =* 0.001	*P =* 0.261
None	22/85 (25.9%)	1	1	1
1	31/64 (48.4%)	2.69 (1.35–5.36)	2.93 (1.45–5.93)	1.79 (0.82–3.90)
2 or more	31/60 (51.7%)	3.06 (1.52–6.17)	4.02 (1.85–8.73)	1.91 (0.78–4.69)
**Violence, perpetrator**		*P =* 0.636	*P =* 0.654	*P =* 0.555
No	92/217 (42.4%)	1	1	1
Yes	5/10 (50.0%)	1.36 (0.38–4.83)	1.34 (0.37–4.81)	1.52 (0.38–6.14)
**Violence, victim**		*P =* 0.960	*P =* 0.990	*P =* 0.585
No	88/205 (42.9%)		1	1
Yes	10/23 (43.5%)	1.02 (0.43–2.44)	1.01 (0.42–2.43)	1.30 (0.51–3.30)
***STIs***				
**HSV-2**		*P <* 0.001	*P <* 0.001	*P <* 0.001
Negative	50/152 (32.9%)	1	1	1
Positive	49/74 (66.2%)	4.00 (2.22–7.20)	4.52 (2.40–8.50)	4.08 (2.03–8.19)
***N*. *gonorrhoeae/C*. *trachomatis***		*P =* 0.044	*P =* 0.044	*P =* 0.768
Negative	84/209 (40.2%)	1	1	1
Positive	18/30 (60.0%)	2.23 (1.02–4.87)	2.23 (1.02–4.88)	1.15 (0.46–2.89)
***T*. *vaginalis***		*P =* 0.989	*P =* 0.944	*P =* 0.287
Negative	96/225 (42.7%)	1	1	1
Positive	6/14 (42.9%)	1.01 (0.34–3.00)	0.96 (0.32–2.91)	0.51 (0.15–1.77)

^1^Number less than 447 due to the number of samples collected for testing for BV.

^2^Sociodemographic variables adjusted for age and in school/working. Behaviour variables adjusted for age, in school/working, and ever drank alcohol. Sexual behaviour and violence variables adjusted for age, in school/working, ever drank alcohol, genital touching, and ever had sex. STI variables adjusted for age, in school/working, ever drank alcohol, genital touching, ever had sex, and HSV-2. Number of lifetime sexual partners was not included for the adjustment with sexual behaviour due to collinearity with ever had sex. Excludes those who preferred not to answer.

^3^Socioeconomic status from a household-level asset index constructed based on ownership of 27 common household items and housing construction, using principal component analysis. Derived from linking the annual health and demographic surveillance site household-level survey in 2015/2016.

^4^Matriculation is the qualification received upon graduating from high school, and is the minimum requirement for entrance to university.

BV, bacterial vaginosis; HSV-2, herpes simplex virus type 2; OR, odds ratio; STI, sexually transmitted infection.

In the subgroup analysis among participants who reported having had sex, there was some evidence that discussing the last partner’s HIV status was associated with not having a curable STI (adjusted OR 0.48; 95% CI 0.23–1.00; S4 Table). There was no evidence that factors included in this subgroup analysis were associated with either HSV-2 infection or diagnosis of BV (S5 and S6 Tables).

## Discussion

We conducted a nested STI survey among young people aged 15 to 24 years in a rural HDSS in KwaZulu-Natal, and found it to be feasible and acceptable. The HDSS provided infrastructure and a sampling frame to carry out a population-based cross-sectional study of STI/BV prevalence. There was a high burden of STIs/BV in this high HIV prevalence setting. Most of the infections were asymptomatic and would not have been identified or treated using national syndromic management guidelines.

**Fig 3:**
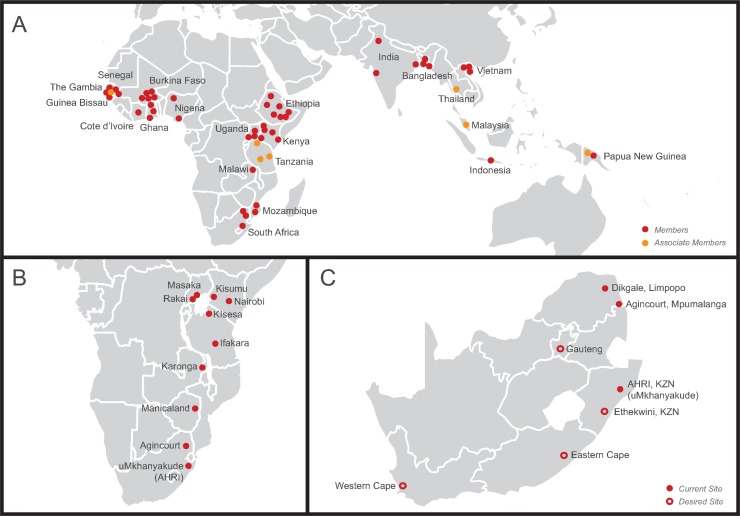
Maps illustrating networks of health and demographic surveillance sites (HDSSs) at the global, regional, and national level. (A) International Network for the Demographic Evaluation of Populations and their Health (INDEPTH Network), a network of 48 members and 7 associate members in 21 low- and middle-income countries in Africa, Asia, and Oceania conducting population-based surveillance of the health status of communities (modified from http://www.indepth-network.org/member-centres). (B) Network for Analysing Longitudinal Population-based HIV/AIDS data on Africa (ALPHA Network), a network of 10 centres in sub-Saharan Africa conducting population-based HIV surveillance (modified from http://alpha.lshtm.ac.uk/partner-study-institutions/). (C) South African Population Research Infrastructure Network (SAPRIN), an expanding network of HDSSs in South Africa (modified from http://saprin.mrc.ac.za/nodes.html). AHRI, Africa Health Research Institute; KZN, KwaZulu-Natal.

This study is a proof of concept that STI surveys can be successfully conducted within HDSS networks such as the INDEPTH Network [12], the Network for Analysing Longitudinal Population-based HIV/AIDS data on Africa (ALPHA Network) [25], the Department of Science and Technology (DST), and the South African Medical Research Council (SAMRC) South African Population Research Infrastructure Network (SAPRIN) [26] (Fig 3). STI surveys can be conducted within the infrastructure of HDSSs with 2 important advantages. First, STI surveys can be carried out in LMICs intermittently to contribute to estimates of the global burden of STIs and to evaluate local implementation of global STI control programmes at a population level. Second, STI surveys can be carried out more frequently in settings with high HIV/STI prevalence to monitor and evaluate enhanced STI/HIV control programmes. HDSS networks could provide a strategic platform to strengthen STI surveillance and control in LMICs, especially in sub-Saharan Africa, where HIV and STI/BV prevalence are high. Importantly, while population-based data are crucial for an effective STI prevention and control programme, these data must be complemented by robust data from high-risk groups (e.g., female sex workers) to account for STI transmission dynamics that depend on high rates of partner change [27].

The prevalence of chlamydia was high in this STI survey among women of both age groups and among men aged 20–24 years. Several studies report high prevalence of chlamydia in South Africa [6,28–31]; both Microbicide Trials Unit (MTN)–003 (VOICE) and HIV Prevention Trials Network (HPTN) 055 showed higher baseline chlamydia prevalence and incidence among women in South Africa compared with other sites in the multi-site studies. Sub-regional or national differences in STI epidemics among young people could be further elucidated in STI surveys in a network of HDSSs. Aetiological diagnosis of STIs is unaffordable and inaccessible for most LMICs. Rapid, accurate, and affordable point-of-care tests might bridge this gap in future [32]. The development of tools such as these must be carried out in parallel with population-based STI surveys and analyses of risk factors.

Although HSV-2 and BV are not curable STIs, better control tools are needed for them, and we recommend continued integration of HSV-2 and BV in STI prevalence surveys. The prevalence of HSV-2 in our study was almost twice as high for young women as for young men, and almost 50% in women aged 20–24 years. Rapid acquisition of HSV-2 after sexual debut has been reported in several studies [8,10], suggesting that HSV-2 seropositivity could be used as a biological proxy for sexual activity. Over 40% of women in this study had BV, consistent with other studies in sub-Saharan Africa [32]. Factors associated with BV in our study (sexual debut, currently having more than 1 sex partner, and HSV-2 infection) are consistent with the literature [32]. Despite BV not being considered a traditional STI, there is an accumulating body of evidence suggesting that sexual transmission is an integral part of its pathogenesis [32]. In addition, BV is associated with serious sequelae, including preterm delivery and increased risk of STI and HIV acquisition and transmission of HIV [3,33–37].

Population-based demographic and behavioural data are also important for planning and evaluating STI prevention and control programmes [38]. In this HIV hyperendemic setting, it is reassuring that there was a higher prevalence of self-reported circumcision among the younger men than among the older men—suggesting the population impact of male medical circumcision programmes. However, the extremely low self-reported condom use at last sex is a tremendous concern. In addition, few participants knew their last partner’s HIV status. In this STI survey, current enrolment in school or working was protective for HSV-2 and BV. These data mirror findings from the AHRI HDSS, which showed that out-of-school youth reported earlier sexual debut and more high-risk sex than in-school youth [39], suggesting that interventions to keep adolescents in school may be just as relevant for other STIs as they are for HIV [40,41].

Strengths of this study include a high rate of acceptability for participation and sample collection, the success in treating those with a curable STI, and the use of a population-based platform as a sampling frame. There are several challenges for carrying out home-based studies, including contacting young people during school hours and the provision of confidential results to participants; however, we maximised contact by modifying the field work hours from 11:00 to 19:00 from Tuesday to Saturday, and provided participants with a choice of mode for receiving results. Once contacted, enrolment into a population-based study of STI/BV testing was acceptable among young people, as was the home-based collection of samples, including the self-collection of genital samples. An additional strength of this study is that it was conducted in an area with persistently high HIV incidence and prevalence. Results of this study could help to inform co‐strategies to address both HIV and STIs that synergise the transmission of HIV.

This study was not without limitations. The sample collection period was limited to 3.5 months by the start of the next HDSS surveillance round, and we did not reach our target of 800 young people. The smaller sample size of 447 provided less precision for prevalence estimates and less power to investigate factors associated with STIs/BV. In addition, the overall coverage in the survey was low, increasing the potential for selection bias. It was challenging to find young men aged 20–24 years at home. HPTN 017 (PopART), a cluster-randomised controlled trial offering home-based HIV counselling and testing in South Africa and Zambia, also reported that young men (32.7%) more often than young women (20.2%) were not at home at the time of visits [42]. Furthermore, many young people were not at home due to migration. The AHRI individual surveys of residents aged 17–49 years indicate that approximately one-fifth of men and women in any survey round have migrated at least once in the last 2 years, and persons with a recent migration history have a higher risk of HIV infection [43]; thus, those with a recent migration history are likely to have a different risk profile. The AHRI HDSS was established in a highly mobile population with a severe HIV epidemic, in which characterisation of migration and mobility was central to its conceptual and data model [44]. Indeed, nesting STI surveys in HDSSs may offer another advantage over one-off de novo STI prevalence surveys: the HDSS sampling frame has information about those who are not enrolled into the study. Additionally, while a one-time survey will miss some of those who have migrated; annual repeat cross-sectional surveys ensure that most age-eligible household members contribute data over time. Reassuringly, STI/BV prevalence weighted for both sampling and non-response data was very similar to the unweighted prevalence or prevalence weighted for sampling only.

Another limitation was that there was evidence of underreporting of sexual behaviours: 6% of participants with a curable STI and 15% of participants with HSV-2 reported never having had sex. Underreporting of sexual behaviour is common, especially among adolescents [45]. We used a computer-assisted survey instrument, study nurses were sex-matched, and interviews were conducted in a private location to improve the completeness and accuracy of self-reported sexual behaviour [16,46], but underreporting was still a challenge. Further research is needed to assess factors affecting the validity of self-reported behaviours among adolescents [47,48]. Importantly, underreporting of sexual behaviour highlights the need to have more robust biological measures of sexual risk, such as STI prevalence.

Finally, this survey is limited to the STIs we tested for—future surveys should consider surveillance of *Mycoplasma genitalium* infection and *N*. *gonorrhoeae* resistance in this population. In addition, surveillance of HPV infection and receipt of vaccination may be important to evaluate implementation of HPV vaccination programmes.

In conclusion, the global population of adolescents and young people is increasing, particularly in sub-Saharan Africa. STIs, including incident HIV, cluster in this population, especially among women. The principles of ‘epidemiology synergy’ between STIs and HIV strongly suggest that STI control must be addressed if HIV is to be brought under effective control [49]. Population-based, representative prevalence estimates of STIs should be complemented by robust prevalence estimates in key populations to gain a full understanding of the burden of STIs and the impact of interventions. Without robust prevalence estimates, moving an international STI agenda forward will continue to be a challenge. Nesting STI prevalence surveys in HDSSs could provide an efficient strategy for obtaining these data.

## Supporting information

S1 STROBE Checklist STROBE statement—Checklist of items that should be included in reports of cross-sectional studies. (DOC)

S1 Analysis Statistical analysis plan for protocol: A study to pilot the surveillance of reproductive tract infections among young people aged 15 to 24 years in the Africa Centre demographic surveillance area. (DOCX)

S1 Table Comparison of selected characteristics of those who enrolled versus those who did not enrol in a population-based STI survey among young people aged 15–24 years in rural KwaZulu-Natal. (DOCX)

S2 Table Contact preferences for results by age and sex in a population-based STI survey among young people aged 15–24 years in rural KwaZulu-Natal among participants who had access to a telephone. (DOCX)

S3 Table Prevalence of STIs in a population-based STI survey among young people aged 15–24 years in rural KwaZulu-Natal: Unweighted prevalence and prevalence with sampling weights only. (DOCX)

S4 Table Factors associated with gonorrhoea, chlamydia, syphilis, and trichomoniasis in a subgroup analysis among individuals who reported having had sex in a population-based STI survey among young people aged 15–24 years in rural KwaZulu-Natal. (DOCX)

S5 Table Factors associated with herpes simplex virus type 2 in a subgroup analysis among individuals who reported having had sex in a population-based STI survey among young people aged 15–24 years in rural KwaZulu-Natal. (DOCX)

S6 Table Factors associated with bacterial vaginosis in a subgroup analysis among women who reported having had sex in a population-based STI survey among young people aged 15–24 years in rural KwaZulu-Natal. (DOCX)

## Abbreviations

AHRI: Africa Health Research Institute
BV: bacterial vaginosis
HDSS: health and demographic surveillance site
HPV: human papillomavirus
HSV-2: herpes simplex virus type 2
INDEPTH Network: International Network for the Demographic Evaluation of Populations and their Health
LMICs: low- and middle-income countries
OR: odds ratio
STI: sexually transmitted infection
TPHA: *Treponema pallidum* haemagglutination
VDRL: Venereal Disease Research Laboratory
