# Supplementary material for: Prevalence of sexually transmitted infections among young people in South Africa: A nested survey in a health and demographic surveillance site
Source: PLoS Med. 2018 Feb 27;15(2):e1002512. doi: 10.1371/journal.pmed.1002512 (PMC5828358; doi:10.1371/journal.pmed.1002512)
Supplement: S1 Analysis — (DOCX) [file pmed.1002512.s002.docx]

**
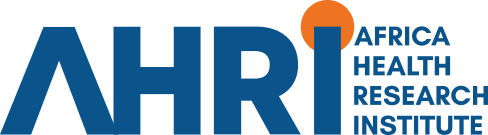
**

**Statistical Analysis Plan**

Protocol Title: A study to pilot the surveillance of reproductive tract infections among young people aged 15 to 24 years in the Africa Centre Demographic Surveillance Area

Study name: Ukuvikela impilo yethu yokuzalana ey**igugu** (English translation: Protecting our precious reproductive health), or the iGugu Study

SAP Version 1.0
25 April 2017

Protocol Version 2.0
16 July 2016

**
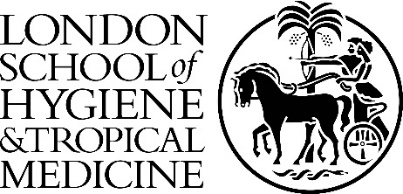
**


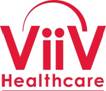

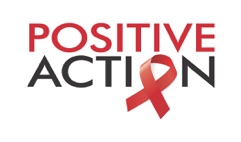

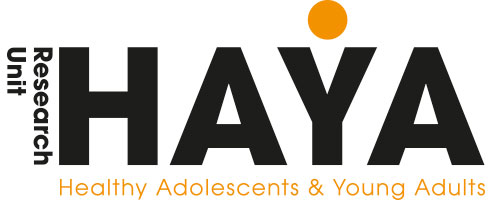


*A partnership of:*


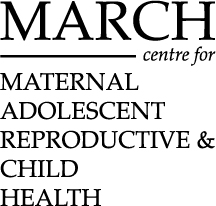


# Table of contents

**Contents**

[1. Aim of the study 3](#_Toc496689777)

[2. Study design 3](#_Toc496689778)

[3. Study population 3](#_Toc496689779)

[4. Sample size 3](#_Toc496689780)

[5. Study objectives 3](#_Toc496689781)

[5.1 Primary objectives 3](#_Toc496689782)

[5.2 Secondary objectives 4](#_Toc496689783)

[6. Objectives for the analysis plan 4](#_Toc496689784)

[7. Study outcomes 4](#_Toc496689785)

[7.1 Feasibility 4](#_Toc496689786)

[7.2 Acceptability 4](#_Toc496689787)

[7.3 Prevalence of reproductive tract infections (RTIs) 4](#_Toc496689788)

[8. Analyses 5](#_Toc496689789)

[8.1 Primary Objective 1-Feasibility 5](#_Toc496689790)

[8.2 Primary Objective 1-Acceptability 5](#_Toc496689791)

[8.3 Primary Objective 2-Prevalence of curable STIs, HSV2 and BV 5](#_Toc496689792)

[8.4 Objective 3: Factors associated with the presence of curable STIs, HSV2 and BV among young men and women 6](#_Toc496689793)

[8.4.1 Descriptive analysis - Baseline characteristics of participants 6](#_Toc496689794)

[8.4.2 Multivariable analysis 7](#_Toc496689795)

[9. References 8](#_Toc496689796)

# 1. Aim of the study

The overall aim of the iGugu Study is to pilot the surveillance of reproductive tract infections (RTIs) among young men and woman aged 15 to 24 years old in the Africa Centre DSA using a 14-week round of the Africa Centre individual surveillance platform as a sampling frame.

# 2. Study design

The study design is cross-sectional of young men and women

# 3. Study population

Young men and women aged 15-24 years living in the Africa Centre DSA

Eligibility criteria for enrollment:

- participants aged 15-24 years at the time of sampling
- resident at the time of the study
- willing to participate and able to provide informed consent

# 4. Sample size

447 young men and women agreed to participate and provided informed consent. Details on sampling methods and intended sample size are provided in the excerpt of the study protocol (Version 1.3, 09 Sept 2016) below:

# 5. Study objectives

## 5.1 Primary objectives

1. Investigate the acceptability and feasibility of population-based sampling of RTI through the individual surveillance platform at the Africa Centre DSA.
2. Estimate the population prevalence of curable STIs (i.e. Chlamydia trachomatis, Neisseria gonorrhoeae, Trichomonas vaginalis, syphilis), HSV-2, *vaginal yeast* and BV (young women only) in the Africa Centre DSA
3. Develop clinical pathways for the treatment of curable RTIs diagnosed through the RTI surveillance platform

## 5.2 Secondary objectives

1. Develop capacity for RTI/STI testing at the Africa Centre Laboratory
2. Store samples for future testing of HPV, vaginal microbiome, metagenomics and transcriptomics (young women only) and other determinants of physiological vulnerability when funding becomes available

# 6. Objectives for the analysis plan

The analysis plan covers two of the study primary objectives:

1. Investigate the acceptability and feasibility of population-based sampling of RTI through the individual surveillance platform at the Africa Centre DSA.
2. Estimate the population prevalence of curable sexually transmitted infections (i.e Chlamydia trachomatis, Neisseria gonorrhoeae, Trichomonas vaginalis, syphilis), HSV-2 and bacterial vaginosis (BV) in the Africa Centre DSA

And the third objective is to:

1. Investigate the risk factors for RTIs among young men and women aged 15-24 years in Africa Centre DSA

# 7. Study outcomes

## 7.1 Feasibility

Feasibility will be measured as the proportion of sampled individuals who were visited and contacted. Reasons for non-contact (e.g. migration) among sampled individuals will also be presented.

## 7.2 Acceptability

Acceptability of RTI sample collection and testing will be measured by the following outcomes:

- proportions of individuals contacted who agreed to participate
- proportions who agreed to each sample collection (e.g. blood, vaginal swabs and urine)
- median and interquartile range of responses to visual analogue scales (VAS) measuring acceptability of sample collection

Variables to be used are described in the next section (Section 1.7).

## 7.3 Prevalence of reproductive tract infections (RTIs)

The following RTIs will be measured:

- Chlamydia trachomatis (Presence/absence)
- Neisseria gonorrhoeae (Presence/absence)
- Trichomonas vaginalis (Presence/absence)
- Syphilis: negative (TP-/VDRL-); early or previously treated infection (TP+/VDRL-); active syphilis (TP+/VDRL+ low titer [<1:8]; TP+/VDRL+ high titer [≥1:8])
- HSV-2 (Presence/absence)
- Bacterial vaginosis (0-3 Normal; 4-6 Intermediate; 7-10 BV)

# 8. Analyses

## 8.1 Primary Objective 1-Feasibility

Feasibility will be examined by sex and age group (15-19 and 20-24 years) using the following outcomes:

- proportion of selected individuals who were visited (regardless of the outcome of visit).
- proportion of visited individuals who were contacted after ≥1 contact attempt

The proportion of those who were not contacted and reasons for failing to contact individuals will also be summarized.

## 8.2 Primary Objective 1-Acceptability

Acceptability of the study will be measured as the proportion of individuals who consented to participate after receiving information about the study and agreed to sample collection (e.g. blood, vaginal swabs and urine). The denominator will include individuals who were selected and contacted.

Acceptability of RTI sample collection among participants will also be measured among individuals who provided samples (swabs or urine), separately for males and females. Medians and interquartile ranges and Box-and-whisker plots will be used to summarize data on the following questions:

- Using a scale 0 to 100 (0 -easy and 100-difficult), how easy or difficult was it to:
- understand the study
- understand instruction for swab (analysis will be restricted to females)
- collect vaginal swab (analysis will be restricted to females)
- understand instruction for urine (analysis will be restricted to males)
- Using a scale 0 to 100 (0-agree and 100-disagree), Disagree or agree with statement about collection of samples:
- felt comfortable
- felt relaxed
- trusted was doing it correctly
- samples taken in a private location
- anxious
- embarrassed

## 8.3 Primary Objective 2-Prevalence of curable STIs, HSV2 and BV

The prevalence (and the 95% confidence interval) of *T. vaginalis*, HSV-2, syphilis, *N. gonorrhoeae*, *C. trachomatis* will be presented overall and separately for males and females and by age group.

Sampling weights will be applied to account for unequal probabilities of selection in the stratified sample design (335 individuals selected from each age group and sex stratum). Sampling weights will be calculated as the inverse probability of selection (i.e. number of individuals sampled in the stratum/ total individuals in the stratum); this will have the effect that participants in the under-represented strata get a larger weight and participants in the over-represented strata get less weights.

In addition, weights will be applied for non-response, calculated as the inverse probability of study participation in strata defined by age group, sex and residence location (urban/peri-urban/rural) using logistic regression. This will be done by combining the data from the entire sampled population with that from the study population, and performing a logistic regression with sex, age group & residence as predictors in the model, to get the predicted probability of participation. The inverse of this predicted probability will be used as the response weight, which will be combined with the sampling weight.

Variables:

- Any curable STIs (1-if diagnosed with at least one STI (chlamydia, gonorrhoeae, trichomoniasis, syphilis) 0-None)
- Chlamydia trachomatis (Presence/absence)
- Neisseria gonorrhoeae (Presence/absence)
- Trichomonas vaginalis (Presence/absence)
- Syphilis: negative (TP-/VDRL-); early or previously treated infection (TP+/VDRL-); active syphilis (TP+/VDRL+ low titer [<1:8]; TP+/VDRL+ high titer [≥1:8])
- HSV-2 (Presence/absence)
- Bacterial vaginosis (Presence/absence)

## 8.4 Objective 3: Factors associated with the presence of curable STIs, HSV2 and BV among young men and women

### 8.4.1 Descriptive analysis - Baseline characteristics of participants

Variables will be summarized by sex and age group. Continuous variables will be summarized using means, standard deviations, medians and interquartile ranges; invalid or impossible values will be set to missing. Frequency counts and percentages will be used to summarize categorical variables.

Variables:

Demographic data

- Age at enrolment (years)
- Level of education completed (primary or less (grade 1-7), secondary (8-11), matric, graduate/postgraduate)
- Working or studying (Working, Studying, Not working or studying)
- Marital status (single, married, separated, living together (not married), civil union)

Alcohol, tobacco and drug use

- Ever smoked cigarette (Yes/No)
- Ever used smokeless products (Yes/No)
- Ever drunk alcohol (Yes/No)
- Drank at least one drink in the past month (Yes, No, Never drinks)
- Drank 5 or more drinks in the past month (Yes, No, Never drinks)
- Ever smoked cannabis (Yes/No)

Circumcision and genital hygiene (restricted to males)

- Circumcised (Yes/No)
- Age at circumcision (Infant, teenager, adult)
- Clean penis with bath soap (Yes/No)

Menstruation and hygiene (restricted to females)

- Menstruation started (Yes/No)
- Age at menarche (years). This will be summarized for individuals who have started menstruating
- Use sanitary pads during menstruation (Yes/No)
- Ever inserted substance in the vagina (Yes/No)
- Ever cleanse inside vagina (Yes/No)

Reproductive health (restricted to females)

- Ever been pregnant (Yes, No, never had sex)
- Age at first pregnancy (years)
- Contraceptive use (Yes/No)
- Contraception method (None, condoms, hormonal, other)

Sexual relationships

- Ever had sex (Yes/No)
- Age at first sex (years)
- In a sexual relationship (Yes/No)
- Used a condom at last sex (Yes/No)
- Drank alcohol before sex (Yes/No)
- Number of people you had sex with in the past 3 months
- Know your partner’s HIV status (Yes/No)

Medical history

- Have current RTI symptoms (Any symptoms, None)

Symptoms will be summarized separately for males and females.

### 8.4.2 Multivariable analysis

Logistic regression models will be used to examine the association between exposure variables and the outcomes. The magnitude of association between exposure and outcome will be summarized using odd ratios and 95% confidence intervals. The model will be built using a conceptual hierarchical framework with three levels: sociodemographic, behavioral and sexual behavior ([1](#_ENREF_1)). Age and sex will be considered a priori confounders and included in all models. First, sociodemographic variables whose age and sex-adjusted association with the outcome is significant at p<0.10 will be added to the model (one at a time). Those that remain significant at p<0.10 will be retained. Behavioral factors will then be added to this model one at a time, and retained if they remain associated at p<0.10. Sexual behavior variables will be handled in the same way. Both crude and adjusted odd ratios will be presented. Many of the sexual behavior variables are only asked of individuals who are sexually active; therefore, the analysis of these variables will be restricted to that sub-group.

# 9. References

1. Victora CG, Huttly SR, Fuchs SC, Olinto MT. The role of conceptual frameworks in epidemiological analysis: a hierarchical approach. International Journal of Epidemiology. 1997;26(1):224-7.
