# Supplementary material for: Prevalence of sexually transmitted infections among young people in South Africa: A nested survey in a health and demographic surveillance site
Source: PLoS Med. 2018 Feb 27;15(2):e1002512. doi: 10.1371/journal.pmed.1002512 (PMC5828358; doi:10.1371/journal.pmed.1002512)
Supplement: S1 Table — (DOCX) [file pmed.1002512.s003.docx]

S1 Table. Comparison of selected characteristics of those who enrolled vs those who did not enrol in a population-based STI survey among young people aged 15-24 years in rural KwaZulu-Natal

|  | **Enrolled  (N=477)** | **Contacted and eligible; refused (N=85)** | **Not contacted (N=390)** | **Not visited (N=171)** | **All who did not enrol (N=895)** |
| --- | --- | --- | --- | --- | --- |
| Age group |  | 0.97^1^ | 0.001^2^ | 0.91^3^ | 0.006^4^ |
| 15-19 | 248 (55.5%) | 47 (55.3%) | 173 (44.4%) | 94 (55.0%) | 425 (47.5%) |
| 20-24 | 199 (44.5%) | 38 (44.7%) | 217 (55.6%) | 77 (45.0%) | 470 (52.5%) |
| Sex |  | 0.17 | <0.001 | <0.001 | <0.001 |
| Male | 188 (42.1%) | 29 (34.1%) | 238 (61.0%) | 101 (59.1%) | 484 (54.1%) |
| Female | 259 (57.9%) | 56 (65.9%) | 152 (39.0%) | 70 (40.9%) | 411 (45.9%) |
| Residence location |  | 0.50 | <0.001 | 0.89 | 0.002 |
| Urban | 19 (4.3 %) | 6 (7.1 %) | 37 (9.5 %) | 7 (4.1 %) | 63 (7.0 %) |
| Peri-urban | 155 (34.7%) | 27 (31.8%) | 93 (23.8%) | 56 (32.7%) | 235 (26.3%) |
| Rural | 273 (61.1%) | 52 (61.2%) | 260 (66.7%) | 108 (63.2%) | 597 (66.7%) |
| Household SES (HDSS)^5^ |  | 0.10 | 0.47 | 0.10 | 0.41 |
| Bottom | 138 (30.9%) | 36 (42.4%) | 131 (33.6%) | 67 (39.2%) | 307 (34.3%) |
| Middle | 152 (34.0%) | 27 (31.8%) | 117 (30.0%) | 55 (32.2%) | 291 (32.5%) |
| Top | 155 (34.7%) | 22 (25.9%) | 136 (34.9%) | 47 (27.5%) | 289 (32.3%) |
| Education (HDSS) |  | 0.87 | 0.02 | 0.15 | 0.009 |
| Primary or less | 43 (9.6 %) | 7 (8.2 %) | 29 (7.4 %) | 19 (11.1%) | 79 (8.8 %) |
| Some secondary | 302 (67.6%) | 56 (65.9%) | 236 (60.5%) | 100 (58.5%) | 541 (60.4%) |
| Matric or above^6^ | 101 (22.6%) | 22 (25.9%) | 125 (32.1%) | 52 (30.4%) | 275 (30.7%) |
| No information | 1 (0.2 %) | 0 | 0 | 0 | 0 |
| HIV status (HDSS) ^7^ |  | 0.41 | 0.46 | >0.99 | 0.52 |
| Positive | 36 (8.1 %) | 4 (4.7 %) | 17 (4.4 %) | 10 (5.8 %) | 44 (4.9 %) |
| Negative | 194 (43.4%) | 34 (40.0%) | 116 (29.7%) | 54 (31.6%) | 277 (30.9%) |

^1^ Chi-squared test comparing those who enrolled and those who were contacted and eligible but refused to participate. ^2^ Chi-squared test comparing those who enrolled and those who had at least one contact attempt but were not found (i.e. not contacted). ^3^ Chi-squared test comparing those who enrolled and those who had no contact attempt (i.e. not visited). ^4^ Chi-squared test comparing those who enrolled and those who were selected but did not enrol.  ^5^Socioeconomic status from a household-level asset index constructed based on ownership of 27 common household items and housing construction, using principal component analysis. ^6^Matriculation (‘matric’) is the qualification received upon graduating from high school, and is the minimum requirement for entrance to university. ^7^Among individuals who participated in the 2016 serosurvey.
