# Supplementary material for: Prevalence of sexually transmitted infections among young people in South Africa: A nested survey in a health and demographic surveillance site
Source: PLoS Med. 2018 Feb 27;15(2):e1002512. doi: 10.1371/journal.pmed.1002512 (PMC5828358; doi:10.1371/journal.pmed.1002512)
Supplement: S2 Table — (DOCX) [file pmed.1002512.s004.docx]

S2 Table. Contact preferences for results by age and sex in population-based STI survey among young people aged 15-24 years in rural KwaZulu-Natal (among individuals who have access to a telephone)

|  | **Male (N=170)** | | | **Female (N=220)** | | | **All participants (N=390)** | | |  |  |  |  |  |  |  |  |  |
| --- | --- | --- | --- | --- | --- | --- | --- | --- | --- | --- | --- | --- | --- | --- | --- | --- | --- | --- |
|  | 15-19 | 20-24 | All | 15-19 | 20-24 | All | 15-19 | 20-24 | All |  |  |  |  |  |  |  |  |  |
| **Preferred method of contact if positive** | | | | | | | | | |  |  |  |  |  |  |  |  |  |
| Telephone call | 65 (59.1%) | 43 (71.7%) | 108 (63.5%) | 56 (58.3%) | 66 (53.2%) | 122 (55.5%) | 121 (58.7%) | 109 (59.2%) | 230 (59.0%) |  |  |  |  |  |  |  |  |  |
| SMS | 42 (38.2%) | 16 (26.7%) | 58 (34.1%) | 35 (36.5%) | 51 (41.1%) | 86 (39.1%) | 77 (37.4%) | 67 (36.4%) | 144 (36.9%) |  |  |  |  |  |  |  |  |  |
| WhatsApp | 3 (2.7 %) | 1 (1.7 %) | 4 (2.4 %) | 5 (5.2 %) | 7 (5.6 %) | 12 (5.5 %) | 8 (3.9 %) | 8 (4.3 %) | 16 (4.1 %) |  |  |  |  |  |  |  |  |  |
| **Preferred method of contact if negative** | | | | | | | | | |  |  |  |  |  |  |  |  |  |
| Telephone call | 66 (60.0%) | 40 (66.7%) | 106 (62.4%) | 52 (54.2%) | 64 (51.6%) | 116 (52.7%) | 118 (57.3%) | 104 (56.5%) | 222 (56.9%) |  |  |  |  |  |  |  |  |  |
| SMS | 41 (37.3%) | 18 (30.0%) | 59 (34.7%) | 39 (40.6%) | 53 (42.7%) | 92 (41.8%) | 80 (38.8%) | 71 (38.6%) | 151 (38.7%) |  |  |  |  |  |  |  |  |  |
| WhatsApp | 3 (2.7 %) | 2 (3.3 %) | 5 (2.9 %) | 5 (5.2 %) | 7 (5.6 %) | 12 (5.5 %) | 8 (3.9 %) | 9 (4.9 %) | 17 (4.4 %) |  |  |  |  |  |  |  |  |  |
