# Supplementary material for: Prevalence of sexually transmitted infections among young people in South Africa: A nested survey in a health and demographic surveillance site
Source: PLoS Med. 2018 Feb 27;15(2):e1002512. doi: 10.1371/journal.pmed.1002512 (PMC5828358; doi:10.1371/journal.pmed.1002512)
Supplement: S3 Table — (DOCX) [file pmed.1002512.s005.docx]

S3 Table. Prevalence of STIs in population-based STI survey among young people aged 15-24 years in rural KwaZulu-Natal: unweighted prevalence and with sampling weights only

|  |  | **Male** |  |  | **Female** |  |  | **All participants** |  |
| --- | --- | --- | --- | --- | --- | --- | --- | --- | --- |
|  | **15**–**19 years** | **20**–**24 years** | **All males** | **15**–**19 years** | **20**–**24 years** | **All females** | **15**–**19 years** | **20**–**24 years** | **All participants** |
|  | **(N=124)** | **(N=64)** | **(N=188)** | **(N=124)** | **(N=135)** | **(N=259)** | **(N=248)** | **(N=199)** | **(N=477)** |
| **Unweighted** |  |  |  |  |  |  |  |  |  |
| Gonorrhoea |  |  |  |  |  |  |  |  |  |
| Positive | 2.4% (0.8-7.3) | 0 | 1.6% (0.5-4.8) | 1.7% (0.4-6.4) | 3.1% (1.2-8.2) | 2.4% (1.1-5.3) | 2.0% (0.8-4.8) | 2.1% (0.8-5.5) | 2.1% (1.1-3.9) |
| Chlamydia |  |  |  |  |  |  |  |  |  |
| Positive | 1.6% (0.4-6.3) | 14.1% (7.4-25.1) | 5.9% (3.3-10.1) | 10.7% (6.3-17.7) | 10.2% (6.0-16.9) | 10.5% (7.2-15.0) | 6.1% (3.7-9.9) | 11.5% (7.7-16.9) | 8.5% (6.2-11.5) |
| T. vaginalis |  |  |  |  |  |  |  |  |  |
| Positive | 0 | 1.6% (0.2-10.5) | 0.5% (0.1-3.7) | 1.7% (0.4-6.4) | 9.4% (5.4-16.0) | 5.6% (3.4-9.3) | 0.8% (0.2-3.2) | 6.8% (4.0-11.3) | 3.4% (2.1-5.6) |
| Syphilis |  |  |  |  |  |  |  |  |  |
| Active | 0 | 0 | 0 | 0 | 0.8% (0.1-5.6) | 0.4% (0.1-3.0) | 0 | 0.5% (0.1-3.8) | 0.2% (0.1-1.7) |
| Recent/previously treated | 0 | 1.6% (0.2-10.7) | 0.5% (0.1-3.8) | 0 | 3.3% (1.2-8.4) | 1.7% (0.6-4.4) | 0 | 2.7% (1.1-6.3) | 1.2% (0.5-2.8) |
| HSV-2 |  |  |  |  |  |  |  |  |  |
| Positive | 12.5% (7.6-19.8) | 22.2% (13.5-34.3) | 15.8% (11.2-21.9) | 17.7% (11.7-25.9) | 48.0% (39.2-56.8) | 33.5% (28.0-39.4) | 15.0% (11.0-20.2) | 39.2% (32.6-46.3) | 25.8% (22.0-29.9) |
| B. vaginosis |  |  |  |  |  |  |  |  |  |
| Intermediate | - | - | - | 9.2% (5.2-16.0) | 11.7% (7.0-18.8) | 10.5% (7.1-15.1) | - | - | - |
| Positive | - | - | - | 41.2% (32.6-50.3) | 44.2% (35.5-53.2) | 42.7% (36.5-49.1) | - | - | - |
| **Sampling weights only** | | | | | | | | | |
| Gonorrhoea |  |  |  |  |  |  |  |  |  |
| Positive | 2.4% (0.8-7.3) | 0 | 1.8% (0.6-5.4) | 1.7% (0.4-6.4) | 3.1% (1.2-8.2) | 2.3% (1.0-5.1) | 2.1% (0.9-4.9) | 2.1% (0.8-5.4) | 2.1% (1.1-4.0) |
| Chlamydia |  |  |  |  |  |  |  |  |  |
| Positive | 1.6% (0.4-6.3) | 14.0% (7.4-25.0) | 5.0% (2.8-8.7) | 10.7% (6.3-17.7) | 10.2% (6.0-16.9) | 10.5% (7.2-15.1) | 6.0% (3.7-9.6) | 11.5% (7.7-16.9) | 8.0% (5.8-10.8) |
| T. vaginalis |  |  |  |  |  |  |  |  |  |
| Positive | 0 | 1.6% (0.2-10.5) | 0.4% (0.1-3.0) | 1.7% (0.4-6.4) | 9.4% (5.4-16.0) | 5.1% (3.0-8.4) | 0.8% (0.2-3.1) | 6.8% (4.0-11.3) | 3.0% (1.8-4.8) |
| Syphilis |  |  |  |  |  |  |  |  |  |
| Active | 0 | 0 | 0 | 0 | 0.8% (0.1-5.6) | 0.4% (0.1-2.6) | 0 | 0.5% (0.1-3.7) | 0.2% (0.1-1.4) |
| Recent/previously treated | 0 | 1.6% (0.2-10.7) | 0.4% (0.1-3.0) | 0 | 3.3% (1.2-8.4) | 1.4% (0.5-3.8) | 0 | 2.7% (1.1-6.3) | 1.0% (0.4-2.3) |
| HSV-2 |  |  |  |  |  |  |  |  |  |
| Positive | 12.5% (7.6-19.8) | 22.2% (13.5-34.3) | 15.2% (10.7-21.1) | 17.7% (11.6-25.9) | 48.0% (39.2-56.8) | 31.2% (25.9-37.1) | 14.9% (10.9-20.1) | 39.1% (32.5-46.1) | 23.8% (20.2-27.9) |
| B. vaginosis |  |  |  |  |  |  |  |  |  |
| Intermediate | - | - | - | 9.2 % (5.2 -16.0) | 11.7% (7.0 -18.8) | 10.3% (7.0 -14.9) | - | - | - |
| Positive | - | - | - | 41.1% (32.6-50.2) | 44.2% (35.5-53.2) | 42.4% (36.2-48.9) | - | - | - |
