# Supplementary material for: Prevalence of sexually transmitted infections among young people in South Africa: A nested survey in a health and demographic surveillance site
Source: PLoS Med. 2018 Feb 27;15(2):e1002512. doi: 10.1371/journal.pmed.1002512 (PMC5828358; doi:10.1371/journal.pmed.1002512)
Supplement: S4 Table — (DOCX) [file pmed.1002512.s006.docx]

S4 Table. Factors associated with chlamydia, gonorrhoea, syphilis and trichomoniasis in a subgroup analysis among individuals who reported having had sex in a population-based STI survey among young people aged 15-24 years in rural KwaZulu-Natal (N=210)

|  | n with Any STI/N(%) | crude OR (95% CI) | Age-sex adj OR (95%CI) | Adjusted OR^1^ (95% CI) |
| --- | --- | --- | --- | --- |
| Genital touching |  | P=0.766 | P=0.899 | P=0.870 |
| No | 13 / 57 (22.8%) | 1 | 1 | 1 |
| Yes | 32 / 153 (20.9%) | 0.90 (0.43 -1.86 ) | 0.95 (0.46 -2.00 ) | 0.93 (0.42 -2.09 ) |
| Oral sex (receive) |  | P=0.637 | P=0.440 | P=0.476 |
| No | 27 / 120 (22.5%) | 1 | 1 | 1 |
| Yes | 17 / 86 (19.8%) | 0.85 (0.43 -1.68 ) | 0.76 (0.38 -1.53 ) | 0.76 (0.36 -1.62 ) |
| Oral sex (provide) |  | P=0.649 | P=0.541 | P=0.443 |
| No | 33 / 146 (22.6%) | 1 | 1 | 1 |
| Yes | 11 / 56 (19.6%) | 0.84 (0.39 -1.80 ) | 0.78 (0.36 -1.71 ) | 0.72 (0.31 -1.68 ) |
| Number of lifetime sexual partners |  | P=0.735 | P=0.600 | P=0.848 |
| One | 16 / 79 (20.3%) | 1 | 1 | 1 |
| Two or more | 23 / 103 (22.3%) | 1.13 (0.55 -2.32 ) | 1.23 (0.57 -2.62 ) | 0.93 (0.42 -2.05 ) |
| Discuss last partner’s HIV status |  | P=0.114 | P=0.060 | P=0.051 |
| No | 26 / 102 (25.5%) | 1 | 1 | 1 |
| Yes | 16 / 98 (16.3%) | 0.57 (0.28 -1.14 ) | 0.50 (0.25 -1.03 ) | 0.48 (0.23 -1.00 ) |
| Condom at last sex |  | P=0.180 | P=0.274 | P=0.291 |
| No | 28 / 111 (25.2%) | 1 | 1 | 1 |
| Yes | 16 / 92 (17.4%) | 0.62 (0.31 -1.24 ) | 0.68 (0.34 -1.36 ) | 0.67 (0.32 -1.40 ) |
| Transactional sex |  | P=0.922 | P=0.934 | P=0.825 |
| No | 38 / 179 (21.2%) | 1 | 1 | 1 |
| Yes | 4 / 18 (22.2%) | 1.06 (0.33 -3.41 ) | 1.05 (0.32 -3.40 ) | 1.15 (0.34 -3.84 ) |
| Violence-perpetrator |  | P=0.597 | P=0.691 | P=0.910 |
| No | 40 / 185 (21.6%) | 1 | 1 | 1 |
| Yes | 2 / 13 (15.4%) | 0.66 (0.14 -3.10 ) | 0.73 (0.15 -3.49 ) | 0.91 (0.18 -4.55 ) |
| Violence-victim |  | P=0.673 | P=0.676 | P=0.803 |
| No | 36 / 177 (20.3%) | 1 | 1 | 1 |
| Yes | 6 / 25 (24.0%) | 1.24 (0.46 -3.32 ) | 1.24 (0.45 -3.37 ) | 1.15 (0.39 -3.40 ) |

^1^Sexual behaviour variables adjusted for age, gender, ever drank alcohol and discuss partner’s HIV status. Excludes those who preferred not to answer.
