# Supplementary material for: Prevalence of sexually transmitted infections among young people in South Africa: A nested survey in a health and demographic surveillance site
Source: PLoS Med. 2018 Feb 27;15(2):e1002512. doi: 10.1371/journal.pmed.1002512 (PMC5828358; doi:10.1371/journal.pmed.1002512)
Supplement: S5 Table — (DOCX) [file pmed.1002512.s007.docx]

S5 Table. Factors associated with Herpes Simplex Virus-2 in a subgroup analysis among individuals who reported having had sex in a population-based STI survey among young people aged 15-24 years in rural KwaZulu-Natal (N=201)

|  | **No. with HSV-2/N(%)** | **crude OR (95% CI)** | **Age-sex adj OR (95%CI)** | **Adjusted OR^1^ (95% CI)** |
| --- | --- | --- | --- | --- |
| Genital touching |  | P=0.317 | P=0.145 | P=0.145 |
| No | 17 / 53 (32.1%) | 1 | 1 | 1 |
| Yes | 59 / 148 (39.9%) | 1.40 (0.72 -2.73 ) | 1.69 (0.83 -3.42 ) | 1.88 (0.80 -4.39 ) |
| Oral sex (receive) |  | P=0.967 | P=0.273 | P=0.351 |
| No | 44 / 115 (38.3%) | 1 | 1 | 1 |
| Yes | 32 / 83 (38.6%) | 1.01 (0.57 -1.81 ) | 0.70 (0.37 -1.33 ) | 0.71 (0.34 -1.46 ) |
| Oral sex (provide) |  | P=0.797 | P=0.305 | P=0.243 |
| No | 52 / 138 (37.7%) | 1 | 1 | 1 |
| Yes | 20 / 56 (35.7%) | 0.92 (0.48 -1.75 ) | 0.69 (0.34 -1.40 ) | 0.62 (0.28 -1.39 ) |
| Number of lifetime partners |  | P=0.119 | P=0.102 | P=0.097 |
| One | 21 / 75 (28.0%) | 1 | 1 | 1 |
| Two or more | 39 / 99 (39.4%) | 1.67 (0.88 -3.19 ) | 1.82 (0.89 -3.74 ) | 1.85 (0.90 -3.83 ) |
| Discussed last partner’s HIV status |  | P=0.758 | P=0.171 | P=0.501 |
| No | 39 / 99 (39.4%) | 1 | 1 | 1 |
| Yes | 35 / 94 (37.2%) | 0.91 (0.51 -1.63 ) | 0.64 (0.34 -1.21 ) | 0.78 (0.39 -1.59 ) |
| Condom at last sex |  | P=0.025 | P=0.154 | P=0.567 |
| No | 48 / 105 (45.7%) | 1 | 1 | 1 |
| Yes | 27 / 90 (30.0%) | 0.51 (0.28 -0.92 ) | 0.63 (0.34 -1.19 ) | 0.81 (0.40 -1.65 ) |
| Transactional sex |  | P=0.055 | P=0.058 | P=0.150 |
| No | 64 / 172 (37.2%) | 1 | 1 | 1 |
| Yes | 11 / 18 (61.1%) | 2.65 (0.98 -7.19 ) | 2.83 (0.97 -8.26 ) | 2.47 (0.72 -8.44 ) |
| Violence-perpetrator^2^ |  | P=0.327 | P=0.432 |  |
| No | 71 / 180 (39.4%) | 1 | 1 | ‒ |
| Yes | 3 / 12 (25.0%) | 0.51 (0.13 -1.96 ) | 0.56 (0.14 -2.35 ) | ‒ |
| Violence-victim |  | P=0.773 | P=0.866 | P=0.789 |
| No | 66 / 171 (38.6%) | 1 | 1 | 1 |
| Yes | 10 / 24 (41.7%) | 1.14 (0.48 -2.71 ) | 1.08 (0.42 -2.78 ) | 0.86 (0.30 -2.52 ) |

^1^Adjusted for age, gender, in school/working and number of lifetime sexual partners. Numbers were very small for violence -perpetrator, so it was automatically removed from the model.
