# Supplementary material for: Prevalence of sexually transmitted infections among young people in South Africa: A nested survey in a health and demographic surveillance site
Source: PLoS Med. 2018 Feb 27;15(2):e1002512. doi: 10.1371/journal.pmed.1002512 (PMC5828358; doi:10.1371/journal.pmed.1002512)
Supplement: S6 Table — (DOCX) [file pmed.1002512.s008.docx]

S6 Table. Factors associated with bacterial vaginosis in a subgroup analysis among individuals who reported having had sex in a population-based STI survey among young people aged 15-24 years in rural KwaZulu-Natal (N=143).

|  | **n with BV/N(%)** | **crude OR (95% CI)** | **Age adj OR (95%CI)** | **Adjusted OR ^1^(95% CI)** |
| --- | --- | --- | --- | --- |
| Genital touching |  | P=0.059 | P=0.062 | P=0.280 |
| No | 18 / 45 (40.0%) | 1 | 1 | 1 |
| Yes | 56 / 98 (57.1%) | 2.00 (0.98 -4.10 ) | 1.99 (0.97 -4.09 ) | 1.52 (0.71 -3.28 ) |
| Oral sex (receive) |  | P=0.900 | P=0.731 | P=0.961 |
| No | 39 / 76 (51.3%) | 1 | 1 | 1 |
| Yes | 33 / 63 (52.4%) | 1.04 (0.53 -2.04 ) | 1.13 (0.57 -2.24 ) | 0.98 (0.48 -2.03 ) |
| Oral sex (provide) |  | P=0.978 | P=0.867 | P=0.829 |
| No | 50 / 98 (51.0%) | 1 | 1 | 1 |
| Yes | 20 / 39 (51.3%) | 1.01 (0.48 -2.12 ) | 1.07 (0.50 -2.27 ) | 0.92 (0.42 -2.02 ) |
| Number of lifetime partners |  | P=0.650 | P=0.288 | P=0.582 |
| One | 29 / 61 (47.5%) | 1 | 1 | 1 |
| Two or more | 31 / 60 (51.7%) | 1.18 (0.58 -2.41 ) | 1.52 (0.70 -3.30 ) | 1.26 (0.55 -2.88 ) |
| Discussed last partner’s HIV status |  | P=0.367 | P=0.290 | P=0.230 |
| No | 31 / 64 (48.4%) | 1 | 1 | 1 |
| Yes | 41 / 73 (56.2%) | 1.36 (0.70 -2.68 ) | 1.45 (0.73 -2.87 ) | 1.55 (0.76 -3.19 ) |
| Condom at last sex |  | P=0.465 | P=0.561 | P=0.633 |
| No | 41 / 82 (50.0%) | 1 | 1 | 1 |
| Yes | 31 / 55 (56.4%) | 1.29 (0.65 -2.57 ) | 1.23 (0.61 -2.46 ) | 1.19 (0.58 -2.46 ) |
| Transactional sex |  | P=0.134 | P=0.124 | P=0.212 |
| No | 64 / 124 (51.6%) | 1 | 1 | 1 |
| Yes | 9 / 12 (75.0%) | 2.81 (0.73 -10.88) | 2.92 (0.75 -11.40) | 2.42 (0.60 -9.67 ) |
| Violence-perpetrator |  | P=0.597 | P=0.697 | P=0.537 |
| No | 67 / 126 (53.2%) | 1 | 1 | 1 |
| Yes | 3 / 7 (42.9%) | 0.66 (0.14 -3.07 ) | 0.73 (0.16 -3.46 ) | 0.57 (0.09 -3.44 ) |
| Violence-victim |  | P=0.399 | P=0.488 | P=0.869 |
| No | 66 / 120 (55.0%) | 1 | 1 | 1 |
| Yes | 7 / 16 (43.8%) | 0.64 (0.22 -1.82 ) | 0.69 (0.24 -1.99 ) | 0.91 (0.30 -2.80 ) |

^1^Adjusted for age and in school/working and ever drunk alcohol. Excludes those who preferred not to answer.
